# A Fast‐Charging and High‐Temperature All‐Organic Rechargeable Potassium Battery

**DOI:** 10.1002/advs.202106116

**Published:** 2022-10-31

**Authors:** Kaiqiang Qin, Kathryn Holguin, Jinghao Huang, Motahareh Mohammadiroudbari, Fu Chen, Zhenzhen Yang, Gui‐Liang Xu, Chao Luo

**Affiliations:** ^1^ Department of Chemistry and Biochemistry George Mason University Fairfax VA 22030 USA; ^2^ Department of Chemistry and Biochemistry University of Maryland College Park MD 20742 USA; ^3^ Chemical Sciences and Engineering Division Argonne National Laboratory Lemont IL 60439 USA; ^4^ Quantum Science and Engineering Center George Mason University Fairfax VA 22030 USA

**Keywords:** all‐organic batteries, fast charge, high temperature, organic electrode materials, rechargeable potassium batteries

## Abstract

Developing fast‐charging, high‐temperature, and sustainable batteries is critical for the large‐scale deployment of energy storage devices in electric vehicles, grid‐scale electrical energy storage, and high temperature regions. Here, a transition metal‐free all‐organic rechargeable potassium battery (RPB) based on abundant and sustainable organic electrode materials (OEMs) and potassium resources for fast‐charging and high‐temperature applications is demonstrated. N‐doped graphene and a 2.8 m potassium hexafluorophosphate (KPF_6_) in diethylene glycol dimethyl ether (DEGDME) electrolyte are employed to mitigate the dissolution of OEMs, enhance the electrode conductivity, accommodate large volume change, and form stable solid electrolyte interphase in the all‐organic RPB. At room temperature, the RPB delivers a high specific capacity of 188.1 mAh g^−1^ at 50 mA g^−1^ and superior cycle life of 6000 and 50000 cycles at 1 and 5 A g^−1^, respectively, demonstrating an ultra‐stable and fast‐charging all‐organic battery. The impressive performance at room temperature is extended to high temperatures, where the high‐mass‐loading (6.5 mg cm^−2^) all‐organic RPB exhibits high‐rate capability up to 2 A g^−1^ and a long lifetime of 500 cycles at 70–100 °C, demonstrating a superb fast‐charging and high‐temperature battery. The cell configuration demonstrated in this work shows great promise for practical applications of sustainable batteries at extreme conditions.

## Graphical abstract

This work demonstrates promising cell configuration for fast‐charging, high‐temperature, and sustainable batteries by employing tetrahydroxy‐1,4‐benzoquinone potassium salt/N‐doped graphene (TBPS/NG) as an anode, polyaniline/N‐doped graphene (PANI/NG) as a cathode, and 2.8 m potassium hexafluorophosphate (KPF_6_) in diethylene glycol dimethy ether (DEGDME) as the electrolyte. The corresponding all‐organic rechargeable potassium batteries exhibit exceptional electrochemical performance in terms of high capacity and superior cycle life.

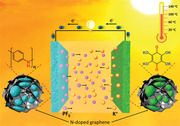

## 1 Introduction

The substantial demands for the transition to a low‐carbon economy have stimulated the development of sustainable batteries for portable electronics, electric vehicles, and grid‐scale electrical energy storage.^[^ ^1^, ^2^, ^3^, ^4^, ^5^ ^]^ However, the state‐of‐the‐art Li‐ion batteries (LIBs), comprising transition metal‐based inorganic electrode materials, cannot satisfy these demands because of the massive carbon dioxide emission from the material fabrication and battery production processes.^[^ ^6^, ^7^ ^]^ To address this challenge, developing green and sustainable battery technologies beyond LIBs and exploiting new structures based on abundant and sustainable organic electrode materials (OEMs) are critical.^[^ ^8^, ^9^, ^10^ ^]^ Among the emerging battery systems beyond LIBs, rechargeable potassium batteries (RPBs) stand out as a promising alternative due to their unique advantages as follows. 1) K resources (2.09% in the Earth's crust) are much more cost‐effective and abundant than Li resources (0.0017% in the Earth's crust), making RPBs promising for sustainable batteries;^[^ ^11^, ^12^, ^13^, ^14^, ^15^, ^16^ ^]^ 2) The standard redox potential of K/K^+^ (−2.924 V) versus the standard hydrogen electrode (SHE) is close to that of Li/Li^+^ (−3.04 V), providing potentially high‐voltage RPBs.^[^ ^17^, ^18^, ^19^, ^20^ ^]^ 3) The ionic conductivity of K‐ion electrolytes is higher than Li‐ion electrolytes due to the weaker Lewis acidity and smaller Stock radius of K‐ions.^[^ ^21^, ^22^, ^23^, ^24^ ^]^ Though the ionic radius of potassium (1.38 Å) is larger than lithium (0.76 Å), the Stock's radius of K^+^ (3.6 Å) is smaller than that of Li^+^ (4.8 Å) in propylene carbonate solvents, indicating much higher ion mobility and ion conductivity.^[^ ^25^, ^26^, ^27^, ^28^, ^29^ ^]^ As has been proved by computational chemistry, the diffusion coefficient of K^+^ is approximately three times higher than that of Li^+^, rendering RPBs promising for fast‐charging batteries.^[^ ^30^, ^31^ ^]^ Therefore, developing high‐performance RPBs is pivotal for next‐generation sustainable energy storage devices.

As is well‐documented, the commercial LIBs not only suffer from low environmental and energy sustainability but also cannot satisfy the demands for fast‐charging (<15 min for full recharge) and high‐temperature (>60 °C) applications.^[^ ^32^, ^33^ ^]^ The development of fast‐charging and high‐temperature RPBs as alternatives to LIBs is essential for the large‐scale applications of energy storage devices in electric vehicles, well drilling industry, and high temperature regions such as deserts and equatorial areas. However, the larger ion size of K^+^ than Li^+^ triggers more severe volume change during the charge and discharge process, resulting in particle pulverization and fast capacity decay in RPBs.^[^ ^34^ ^]^ Moreover, the fast‐charging and high‐temperature conditions enhance the ion/electron transfer in the electrode materials, further compromising the structural integrity of the electrodes and solid‐electrolyte interphase (SEI). Hence, most inorganic materials, which exhibit excellent electrochemical performance in LIBs, do not extend their high capacity and high cyclic stability to RPBs.^[^ ^35^ ^]^ To obtain high‐performance, fast‐charging, and high‐temperature RPBs, lightweight, abundant, flexible, and tunable OEMs offer opportunities. Due to the abundant structural diversity and tunability of organic materials, OEMs can function as both cathodes and anodes in RPBs to achieve high‐performance all‐organic batteries via rational structure design and performance optimization.^[^ ^36^, ^37^, ^38^ ^]^ The high flexibility and high thermal stability of OEMs not only accommodate the large‐size K^+^ during fast charge/discharge but also enable the reversible and stable high‐temperature potassiation/de‐potassiation process.

Considerable efforts have been devoted to developing advanced RPBs at ambient temperature and high temperature. Though there are some reports of RPBs operating at 50–60 °C,^[^ ^39^, ^40^, ^41^ ^]^ the demonstrations of RPBs at temperatures above 65 °C are very few due to the low melting point of metallic K (63.65 °C) and unstable electrolytes/SEI at high temperatures. In contrast, all‐organic RPBs are ideal candidates for high‐temperature RPBs due to their advantages as follows: 1) The all‐organic RPBs based on p‐type organic cathodes and n‐type organic anodes do not require metallic K, so the working temperature is not limited by the melting point of metallic K; 2) Most OEMs are thermally stable at the temperatures below 200 °C;^[^ ^42^ ^]^ 3) The high flexibility of OEMs retains the structural integrity during fast ion/electron transfer at high temperatures.^[^ ^43^ ^]^ In addition to organic electrodes, electrolytes also play a vital role in high‐temperature RPBs.^[^ ^44^, ^45^ ^]^ The gassing of volatile organic solvents in the commercial electrolytes and the instability of SEI at high operating temperatures can result in the failure of rechargeable batteries. To overcome these challenges, high‐boiling‐point or nonvolatile solvents are required for organic electrolytes. More importantly, a robust and thermally stable SEI derived from organic electrolytes is also required for high‐temperature batteries. Therefore, developing advanced OEMs and organic electrolytes are crucial for fast‐charging and high‐temperature RPBs.

**Figure 1:**
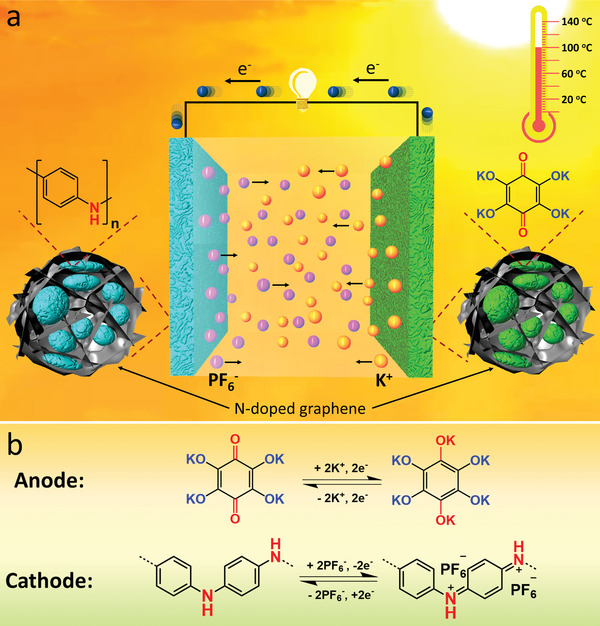
Schematic illustration and redox reactions in the all‐organic battery. a) The schematic of the all‐organic rechargeable potassium battery; b) Redox reactions in the organic anode and cathode during charge/discharge.

Herein, we present a fast‐charging and high‐temperature all‐organic RPB using tetrahydroxy‐1,4‐benzoquinone potassium salt/N‐doped graphene (TBPS/NG) as an anode and polyaniline/N‐doped graphene (PANI/NG) as a cathode. In the all‐organic PRB, the highly conductive NG is used in both the cathode and the anode to increase the conductivity, accommodate the large volume change, and retain the structural integrity of organic electrodes during repeated potassiation/de‐potassiation processes. A highly concentrated electrolyte, 2.8 m KPF_6_ in diethylene glycol dimethyl ether (DEGDME), is used to further improve the performance of the all‐organic RPB due to the high boiling point (162 °C) and low volatility of DEGDME, as well as the formation of a stable K_2_CO_3_− and KF‐rich SEI. The stable electrolyte and SEI are of paramount importance for the stability of the fast‐charging and high‐temperature all‐organic RPB. Additionally, the highly concentrated electrolyte mitigates the dissolution of OEMs, further improving long‐term cyclic stability. Consequently, the all‐organic RPB (**Figure** **1**a) delivers impressive electrochemical performance under fast‐charging (up to 10 A g^−1^) and high‐temperature (up to 100 ^o^C) conditions. The reversible redox reactions (Figure 1b) of the carbonyl‐based anode and amine‐based cathode in the all‐organic RPB enable the excellent performance at extreme conditions. At low mass loading of 1.8 mg cm^−2^, the all‐organic RPB delivers a high specific capacity of 188.1 mAh g^−1^ at 50 mA g^−1^ and room temperature. At the high current density of 1 and 5 A g^−1^, superior cycle lives of 6000 and 50 000 cycles are achieved, respectively, demonstrating an ultra‐stable all‐organic RPB. The exceptional electrochemical performance is retained at high mass loading of 6.5 mg cm^−2^ and high temperatures. The high‐mass‐loading all‐organic RPB exhibits fast‐charging capability up to 2 A g^−1^ and a long lifetime of 500 cycles at ≈70–100 °C, demonstrating a superb fast‐charging and high‐temperature battery. Therefore, this work developed promising cell configuration based on abundant OEMs and potassium resources for fast‐charging and high‐temperature sustainable energy storage.

## 2 Results and Discussion

### 2.1 The Structure of TBPS

**Figure 2:**
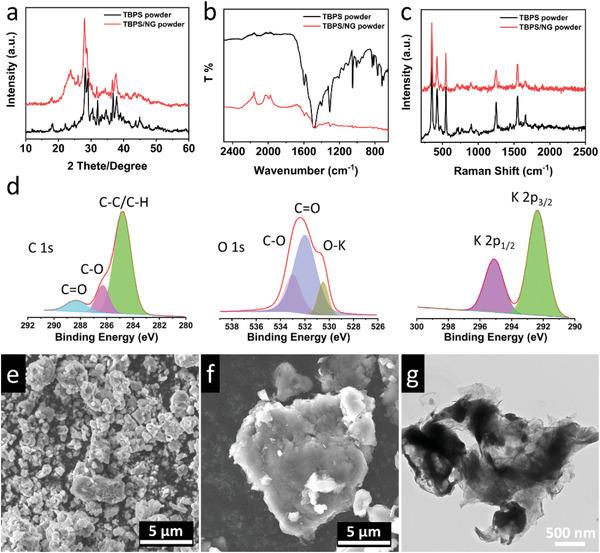
Material characterizations for TBPS and TBPS/NG powders. a) XRD patterns, b) FTIR, and c) Raman spectra of TBPS and TBPS/NG powders; d) XPS C 1s, O 1s, K 2p spectra of the TBPS powder; SEM images of e) TBPS and f) TBPS/NG powders; g) The TEM image of the TBPS/NG powder.

To achieve the all‐organic RPB, we coupled a carbonyl‐based n‐type organic anode with an amine‐based p‐type organic cathode (polyaniline, PANI). The anode material, tetrahydroxy‐1,4‐benzoquinone potassium salt (TBPS), was synthesized by neutralizing tetrahydroxy‐1,4‐benzoquinone with potassium hydroxide in an ethanol solution. The collected TBPS exists as brown powder and turn to black after ball milling with NG (Figure S1, Supporting Information). The carbonyl groups in TBPS are redox centers for K^+^ insertion/extraction, providing a theoretical capacity of 165.43 mAh g^−1^. The design of organic salts is an effective method to suppress the dissolution of organic compounds in the electrolytes due to the formation of ionic bonding and enhanced polarity.^[^ ^46^, ^47^ ^]^ To validate the molecular structure of TBPS, the nuclear magnetic resonance (NMR) was employed using D_2_O as the solvent (Figure S2, Supporting Information). In ^1^H NMR, there is no obvious peak except a singlet peak for the solvent (D_2_O, 4.784 ppm), indicating the complete deprotonation of the tetrahydroxy‐1,4‐benzoquinone precursor. The ^13^C NMR confirms C atoms in two different chemical environments. The peaks at 176.44 and 173.02 ppm represent C atoms connect to potassium oxide groups and the C atoms in carbonyl groups, respectively.^[^ ^39^ ^]^ The molecular and crystalline structure of TBPS were further elucidated by X‐ray diffraction (XRD), Fourier‐transform infrared spectroscopy (FTIR), Raman spectrum, and X‐ray photoelectron spectroscopy (XPS). The sharp peaks in the XRD pattern indicate that TBPS shows high crystallinity (**Figure** **2**a). The intense absorption peaks at ≈1594 cm^−1^ and ≈1052 cm^−1^ in the FTIR spectrum represent the stretching vibrations of C=O and C—O groups, respectively (Figure 2b).^[^ ^12^ ^]^ The two peaks located at 353.9 and 422.3 cm^−1^ in the Raman spectrum (Figure 2c) represent the stretching vibration of O–K groups. The Raman peaks at 1450, 1500, and 1550 cm^−1^ represent C=C and C—C stretching of the benzenoid rings, while the peak at 1661.5 cm^−1^ is ascribed to the stretching vibration of C=O groups.^[^ ^48^ ^]^ In the XPS spectra (Figure 2d), all the peaks were calibrated by the C—C peak in C 1s spectrum at 284.8 eV. The XPS peaks at ≈530.4, 532, and 533 eV in O 1s spectrum are assigned to K—O, C=O, and C—O groups in TBPS.^[^ ^49^ ^]^ The morphological structure of TBPS was measured by scanning electron microscopy (SEM) and transmission electron microscopes (TEM). As shown in Figure 2e and Figure S3a,b, Supporting Information, TBPS powders consist of irregularly shaped and micro‐sized particles. The elemental mapping of TBPS indicates that C, O, and K are uniformly distributed in the compound (Figure S4, Supporting Information). Meanwhile, the TBPS contains ≈0.48 at% F element, which is the impurity from the tetrahydroxy‐1,4‐benzoquinone hydrate precursor (Figure S5, Supporting Information). To improve the electrochemical performance in RPBs, TBPS was mixed with NG by ball milling. The high conductivity, high stability, and high Young's modulus of NG not only enhance the electronic conductivity but also stabilize the TBPS in RPBs.^[^ ^50^, ^51^ ^]^ As shown in Figure 2a–c and Figure S6, Supporting Information, the crystalline and molecular structure of TBPS is retained in the resulting TBPS/NG composite. SEM and TEM images in Figure 2f,g, and Figure S3e–h, Supporting Information, demonstrate that the micro‐sized TBPS particles are fully coated by NG after ball milling. The SEM elemental mapping of TBPS/NG indicates that the C, N, O and K are uniformly distributed in the composite (Figure S7, Supporting Information). These material characterizations confirm the crystalline, molecular, and morphological structure of the devised organic anode materials, TBPS and TBPS/NG composite.

### 2.2 Electrochemical Performance of the TBPS/NG Anode in RPBs

**Figure 3:**
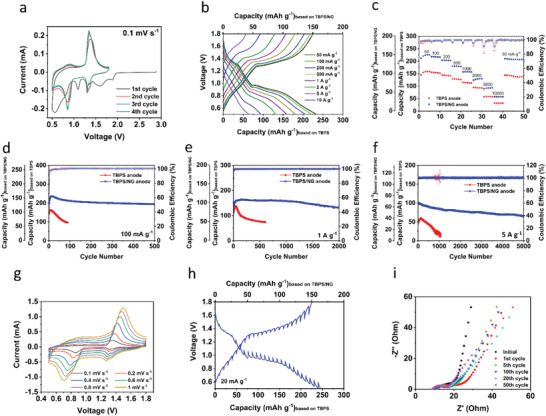
Electrochemical performance of TBPS/NG and TBPS anodes in RPBs at room temperature. a) Cyclic voltammograms at 0.1 mV s^−1^ and b) charge/discharge curves of the TBPS/NG anode at various current densities; c) Rate performance comparison of TBPS/NG and TBPS anodes at various current densities; charge capacities and Coulombic efficiency measured at d) 100 mA g^−1^, e) 1 A g^−1^, and f) 5 A g^−1^ of TBPS/NG and TBPS anodes; g) Cyclic voltammograms at various scan rates; h) Potential response during GITT measurements; i) Impedance analysis before and after charge/discharge.

The electrochemical performance of the TBPS/NG anode in RPBs was investigated by coupling it with the K metal as the counter electrode and using 2.8 m KPF_6_ in DEGDME as the electrolyte. The galvanostatic charge/discharge curves of the TBPS/NG anode at 50 mA g^−1^ show that the initial Coulombic efficiency (CE) is 46.65% due to the formation of SEI, but it quickly enhances to ≈100% after a few cycles (Figure S8a, Supporting Information). The TBPS/NG anode exhibits two pairs of redox plateaus centered at ≈1.3 V and 0.9 V with a reversible specific capacity of 225.5 mAh g^−1^ based on the mass of TBPS. The reversible capacity of the TBPS/NG anode is higher than the theoretical capacity of TBPS because of the capacity contribution by NG. The NG electrodes with two different weight ratios between NG and carbon black were prepared to investigate its electrochemical performance more objectly. The first NG electrode (named NG‐I) was prepared by mixing NG and sodium alginate to form a slurry with the weight ratio of 90:10. To avoid the restacking of NG, the second NG electrode (named NG‐II) was prepared by mixing NG, carbon black, and sodium alginate to form a slurry with the weight ratio of 60:30:10. As shown in Figure S9a, Supporting Information, NG‐I electrode shows a specific capacity of 74.5 mAh g^−1^ at 50 mA g^−1^ in the cutoff window between 0.5 and 1.8 V, and there is no obvious changes for NG‐II electrode (Figure S9c, Supporting Information). The TBPS anode without NG delivers a lower reversible capacity but similar redox plateaus as the TBPS/NG anode (Figure S8b, Supporting Information). In cyclic voltammograms (**Figure** **3**a), two cathodic peaks at 1.31/0.86 V and two anodic peaks at 0.89/1.35 V are observed for the TBPS/NG anode, corresponding to the charge/discharge plateaus in Figure S8a, Supporting Information. This indicates a two‐step reaction between two carbonyl groups and two potassium ions (Figure 1b). As shown in Figure 3b,c, the TBPS/NG anode shows excellent rate capability at room temperature, delivering the specific capacity of 225.5, 218.4, 203.7, 180.4, 157.6, 129.1, 92.7, and 57.8 mAh g^−1^ at the current densities of 50, 100, 200, 500, 1000, 2000, 5000, and 10 000 mA g^−1^ based on the mass of TBPS. After the current density reduces back to 50 mA g^−1^, the specific capacity recovers to 210.4 mAh g^−1^ immediately, demonstrating robust reaction kinetics. In contrast, the TBPS anode without NG shows moderate rate capability, providing a specific capacity of 160.3 mAh g^−1^ at 50 mA g^−1^ and retaining 31.7 mAh g^−1^ at 10 A g^−1^. In addition to the exceptional rate capability, the TBPS/NG anode also displays superior cyclic stability at room temperature. At the low current density of 100 mA g^−1^, the TBPS/NG anode provides an initial reversible capacity of 175.7 mAh g^−1^ and gradually increases to 230.9 mAh g^−1^ after a few cycles. The reversible specific capacity of 192.6 mAh g^−1^ is retained after 500 cycles (Figure 3d). At high current densities of 1 and 5 A g^−1^, the TBPS/NG anode retains reversible capacities of 131.8 mAh g^−1^ after 2000 cycles (Figure 3e) and 65.2 mAh g^−1^ after 5000 cycles (Figure 3f), respectively, corresponding to very slow capacity decay rates of 0.0098% and 0.0071% per cycle. On the contrary, the TBPS anode without NG exhibits poor cycling stability at 100 mA g^−1^, 1 A g^−1^, and 5 A g^−1^, demonstrating the importance of NG to the cycle life of the organic anodes. Figure S10a, Supporting Information, shows the comparison of the cyclic stability of TBPS/NG anode in the KPF_6_ in DEGDME electrolytes with different concentrations. The cyclic stability of the TBPS/NG anode improves with the increased salt concentration in the electrolyte, because the highly concentrated electrolyte effectively mitigates the dissolution of TBPS.

A three‐electrode system (Figure S10b, Supporting Information) was employed for the investigation of the redox potential of K in the electrolyte of 2.8 m KPF_6_ in DEGDME using a stainless steel sheet as the working electrode, K metal as the counter electrode, and a Ag/Ag^+^ nonaqueous electrode (filled with 10 mm AgNO_3_ in CH_3_CN) as the reference electrode. The CV curve which was tested under scan rate of 0.1 mV s^−1^ at the voltage range of −3.8–0 V (versus Ag/Ag^+^) shows that the K plating potential in the 2.8 m KPF_6_ in DEGDME electrolyte is −3.48 V (versus Ag/Ag^+^) in Figure S10c, Supporting Information. The literature value for Ag/AgNO_3_ in CH_3_CN (10 mm) versus Ag/AgCl (filled with saturated KCl) is 345 mV.^[^ ^52^ ^]^ The standard electrode potential of Ag/AgCl (filled with saturated KCl) (*E* ^0^_Ag/AgCl_) against standard hydrogen electrode (SHE) is 0.2224 V. Hence, the standard electrode potential of Ag/Ag^+^ nonaqueous electrode (filled with 10 mm AgNO_3_ in CH_3_CN) ($E_{\mathrm{Ag}/{\mathrm{Ag}}^{+}}^{0}$) was calculated using the formula below:

(1) $$E_{\mathrm{Ag}/{\mathrm{Ag}}^{+}}^{0}=E_{\mathrm{Ag}/\mathrm{AgCl}}^{0}+E_{\mathrm{Difference}}$$

where *E*_Difference_ is the value for Ag/AgNO_3_ in CH_3_CN (10 mm) frit versus Ag/AgCl (filled with saturated KCl). The redox potential of K in the electrolyte of 2.8 m KPF_6_ in DEGDME against SHE (*E_K_*_versus_ *_SHE_*) was calculated using the formula below:

(2) $$E_{K\;vs\;SHE}=E_{\mathrm{Ag}/{\mathrm{Ag}}^{+}}^{0}+E_{Kvs.\;Ag/Ag^{+}}$$

Hence, the *E*_*K* *vs* *SHE*_ in the 2.8 m KPF_6_ in DEGDME electrolyte is −2.9126 V, which is quite close to the *E^0^* of K (−2.924 V).

To further investigate the reaction kinetics of the TBPS/NG anode in RPBs, cyclic voltammetry (CV), galvanostatic intermittent titration technique (GITT), and electrochemical impedance spectroscopy (EIS) were employed. As shown in Figure 3g, the TBPS/NG anode was cycled at various scan rates from 0.1 to 1 mV s^−1^. The anodic peaks shift to a higher potential and the cathodic peaks migrate to a lower potential with the elevated scan rates due to the increased polarization.^[^ ^54^ ^]^ The liner functions are used to analyze the relationship between the peak current and scan rate, which is displayed in Figure S11, Supporting Information. The slope (b) values of anodic and cathodic peaks are 0.7773 and 0.7791, respectively, demonstrating that the reaction kinetics of the TBPS/NG anode is largely contributed by the surface reaction mechanism and exhibits a partial capacitive behavior.^[^ ^55^ ^]^ In addition, GITT curves at the current density of 20 mA g^−1^ (Figure 3h) indicate that the charge and discharge overpotentials are only 45 and 66 mV, respectively, further demonstrating fast kinetics of the TBPS/NG anode.^[^ ^56^ ^]^ The reaction kinetics is also investigated by EIS (Figure 3i). The interfacial impedance of the pristine TBPS/NG anode, represented by the depressed semi‐circle, is ≈10 Ω and increases to ≈26 Ω after the first cycle due to the formation of the SEI layer. The interfacial impedance gradually decreases to ≈10 Ω after five cycles and retains a similar value after 50 cycles, indicating the high stability of the SEI layer. The interfacial impedance is the sum of SEI layer resistance, particle‐to‐particle resistance, and the charge transfer resistance. The stable and robust SEI layer is critical for the stability of the TBPS/NG anode upon long‐term cycling. The electrochemical results validate the outstanding reaction kinetics and high cyclic stability of the TBPS/NG anode in RPBs.

### 2.3 Characterization of the TBPS/NG Anode after Cycling

To investigate the structure stability of the TBPS/NG anode, the morphology of the pristine electrode and cycled electrodes at different current densities were characterized. As shown in Figure S12, Supporting Information, the TBPS/NG in the pristine electrode shows a micro‐sized particle structure and the surface is smooth due to the uniform NG coating. After 20 cycles at the current density of 200 mA g^−1^, abundant NG wrinkles are formed on the surface due to the large volume change during the charge/discharge process (yellow circle area in Figure S13, Supporting Information). The elemental mappings show that C, N, O, F, P, and K are homogeneously distributed in the anode after 20 cycles, indicating uniform formation of the SEI layer (Figure S14, Supporting Information). With the increased current density, more surface wrinkles can be observed in SEM images (Figure S15 and S16, Supporting Information), but the TBPS/NG composite retains the structural integrity even at the high current density of 10 A g^−1^, attributing to the high Young's modulus of NG.^[^ ^57^ ^]^ In contrast, for the TBPS anode without NG (Figures S17 and S18, Supporting Information), obvious cracks and holes were generated on the micro‐sized TBPS particles and the anodes after 20 cycles at 200 mA g^−1^. These results demonstrate that NG can effectively accommodate the large volume changes and retain the structural integrity of the TBPS/NG anode during the repeated charge/discharge processes even at high current densities, endowing the TBPS/NG anode excellent cycling stability.

The surface information and SEI structure of pristine and cycled TBPS/NG anodes were further characterized by XPS (Figure S19, Supporting Information). In the pristine TBPS/NG anode, the XPS peaks at 289 eV in the C 1s spectrum and 534 eV in the O 1s spectrum are ascribed to C and O signals of carboxylate groups, which come from the sodium alginate binder.^[^ ^58^ ^]^ The XPS peaks in F 1s spectrum of the pristine TBPS/NG anode (Figure S19a, Supporting Information) are attributed to the impurities of tetrahydroxy‐1,4‐benzoquinone hydrate precursor, which is confirmed by the element mapping results of tetrahydroxy‐1,4‐benzoquinone hydrate precursor and TBPS powder (Figures S4 and S5, Supporting Information). In the cycled TBPS/NG anode (Figure S19b–d, Supporting Information), the peak at 290 eV in the C 1s XPS spectrum is assigned to –CO_3_^2−^, which indicates the presence of K_2_CO_3_ in the SEI layer.^[^ ^59^ ^]^ In the F 1s spectrum, XPS peaks at 688 and 686 eV are assigned to C—F and P—F, respectively. The XPS peak at 684.3 eV is assigned to K—F, indicating the formation of KF in the SEI layer. In the P 2p spectrum, the XPS peaks at 135 and 138 eV are assigned to P—O and P—F, respectively.^[^ ^12^, ^60^ ^]^ The XPS results prove that K_2_CO_3_, KF, organic fluorides, and phosphates are the main components in the SEI layer. As further indicated by XPS (Figure S19c,d, Supporting Information), the SEI structure in the TBPS/NG anode is retained even after cycling at high current densities of 5 A g^−1^ and 10 A g^−1^, demonstrating that the K_2_CO_3_‐ and KF‐rich SEI is stable and robust under the fast‐charging conditions.

### 2.4 Electrochemical Performance of All‐Organic RPB at Room Temperature

Since the TBPS/NG anode exhibits superior electrochemical performance in RPBs, we couple it with a p‐type organic cathode, PANI, to explore the full cell performance of all‐organic batteries. The low‐cost, abundant, highly conductive, and thermally stable PANI undergoes an anion‐insertion mechanism in rechargeable batteries, providing a high redox potential above 3 V.^[^ ^9^, ^61^ ^]^ In this work, PANI is used in the cathode after ball milling with NG to further improve the performance of the cathode. As shown in the SEM images (Figure S20, Supporting Information), PANI consists of micro‐sized particles, and PANI particles are fully coated by NG and retain the micro‐sized structure after ball milling. To match the capacity with the TBPS/NG anode for the full cell measurements, the electrochemical performance of the PANI/NG cathode is investigated. The PANI/NG cathode exhibits a pair of redox plateaus at ≈3.25 V. At the current density of 100 mA g^−1^, the initial specific capacity of the PANI/NG cathode is 55.2 mAh g^−1^ based on the mass of PANI, and it gradually increases to 195.4 mAh g^−1^ after a few cycles and retains at 174.2 mAh g^−1^ after 600 cycles, demonstrating high specific capacity and good cycling stability (Figure S21, Supporting Information).

**Figure 4:**
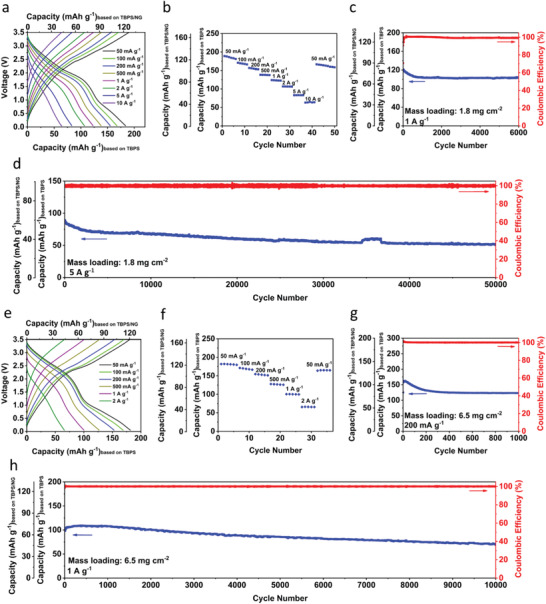
The electrochemical performance of the all‐organic RPB with low mass loading at room temperature. a) Charge/discharge curves at different rates; b) Rate performance at various current densities; Discharge capacities and Coulombic efficiency measured at c) 1 A g^−1^ and d) 5 A g^−1^. The electrochemical performance of the all‐organic RPB with high mass loading at room temperature. e) Charge/discharge curves at different C‐rates. f) Rate performance at various current densities. Discharge capacities and Coulombic efficiency measured at g) 200 mA g^−1^ and h) 1 A g^−1^.

The all‐organic RPB based on the TBPS/NG anode, PANI/NG cathode, and a 2.8 m KPF_6_‐DEGDME electrolyte is tested with both low anode mass loading of 1.8 mg cm^−2^ and high anode mass loading of 6.5 mg cm^−2^ based on the mass of TBPS. **Figure** **4** shows the electrochemical performance of the all‐organic RPB at room temperature. The current densities and capacity of the full cell are calculated based on the mass of TBPS. As shown in Figure 4a,b, the all‐organic RPB with low mass loading of 1.8 mg cm^−2^ exhibits superior rate capability, delivering the specific capacity of 188.1, 169.5, 156.1, 138.4, 123.8, 106.7, 83.1, and 64.9 mAh g^−1^ at the current densities of 50, 100, 200, 500, 1000, 2000, 5000, and 10 000 mA g^−1^. The specific capacity retains at 167.4 mAh g^−1^ after the current density reduces back to 50 mA g^−1^. In cyclic voltammograms at 0.1 mV s^−1^ (Figure S22a, Supporting Information), two cathodic peaks at 1.93 and 0.82 V, and two anodic peaks at 2.6 and 2.87 V are observed, corresponding to the charge/discharge plateaus in Figure 4a. With elevated scan rates (Figure S22b, Supporting Information), the CV peaks shift away from each other due to the enhanced polarity at high currents. Furthermore, the all‐organic RPB delivers excellent cyclic stability and fast charging capability at room temperature. At 1 A g^−1^, the all‐organic RPB delivers an initial reversible capacity of 121.3 mAh g^−1^ and retains at 104 mAh g^−1^ after 6000 cycles, corresponding to a very slow capacity decay rate of 0.0024% per cycle (Figure 4c). Even at 5 A g^−1^, the all‐organic RPB still delivers an initial specific capacity of 89.3 mAh g^−1^ and retains a reversible capacity of 51.9 mAh g^−1^ after 50 000 cycles (Figure 4d), representing the fast kinetics and best cycle life in RPBs. The exceptional electrochemical performance demonstrates that this all‐organic RPB is promising for sustainable and fast‐charging energy storage. Since the all‐organic RPB with low mass loading shows impressive performance, we further exploit the electrochemical performance of the all‐organic RPB with high mass loading of 6.5 mg cm^−2^ (based on TBPS). As shown in Figure 4e,f, the all‐organic RPB with high mass loading retains the outstanding rate capability at room temperature, delivering the specific capacity of 181.5, 168.8, 152.9, 126.8, 100.2 and 65.7 mAh g^−1^ at the current density of 50, 100, 200, 500, 1000, and 2000 mA g^−1^. The specific capacity recovers to 165.1 mAh g^−1^ immediately after the current density reduces back to 50 mA g^−1^. Meanwhile, the all‐organic RPB with high mass loading also delivers high cyclic stability and fast‐charging capability at room temperature. At 200 mA g^−1^, it provides an initial reversible capacity of 157.7 mAh g^−1^ and remains at 123.2 mAh g^−1^ after 1000 cycles (Figure 4g). At 1 A g^−1^, it delivers an initial specific capacity of 102.6 mAh g^−1^ and provides capacity retention of 72.8% after 10 000 cycles (Figure 4h). The exceptional performance of the high‐mass‐loading all‐organic RPB at room temperature renders it promising for practical applications in the sustainable and fast‐charging energy storage field.

### 2.5 Electrochemical Performance of the All‐Organic RPB at High Temperatures

**Figure 5:**
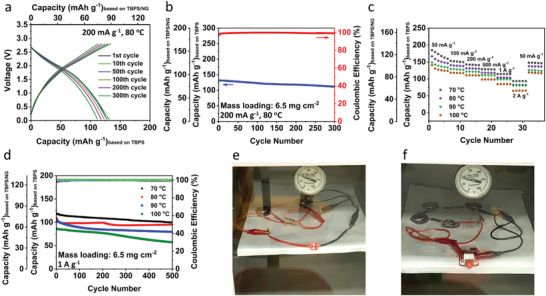
The electrochemical performance of the all‐organic RPB with high mass loading at high temperatures. a) Selected charge/discharge curves and b) cycling stability of the all‐organic RPB at 80 °C under the current density of 200 mA g^−1^. c) Rate capability of the all‐organic RPB at high temperatures. d) Cycling stability of the all‐organic RPB at high temperatures under the current density of 1 A g^−1^. e) Two red LED lights and f) a small fan powered by all‐organic RPBs at ≈90 °C.

Since the all‐organic RPB exhibits impressive performance at room temperature, we further investigate its feasibility at high temperatures. The cyclic stability of the all‐organic RPB with high anode mass loading of 6.5 mg cm^−2^ is assessed at 80 °C. As shown in **Figure** **5**a, the galvanostatic charge/discharge curves at 200 mA g^−1^ show a pair of sloping plateaus centered at 2 V with an initial capacity of 131.8 mAh g^−1^. In the long‐term cycling test, a reversible capacity of 111.3 mAh g^−1^ is retained after 300 cycles (Figure 5b), demonstrating high durability at 80 °C. In addition, the all‐organic RPB with high mass loading also exhibits superior rate capability at high temperatures from 70–100 °C. At 70 °C, the batteries deliver specific capacities of 173.1, 153.5, 142.1, 128.2, 114.3, and 93.5 mAh g^−1^ at the current densities of 50, 100, 200, 500, 1000, and 2000 mA g^−1^ and remains at 148.2 mAh g^−1^ after the current density reduces back to 50 mA g^−1^ (Figure 5c and Figure S23a, Supporting Information). At even higher temperatures, the all‐organic RPB still retains the exceptional rate capability that reversible capacities of 81.8, 80.5, 65.1 mAh g^−1^ at high current density of 2 A g^−1^ are achieved at 80, 90, and 100 °C (Figure 5c and Figure S23b–d, Supporting Information), demonstrating the fast‐charging capability at high temperatures. Hence, the cyclic stability of the all‐organic RPB at high temperatures is further investigated at the high current density of 1 A g^−1^. As shown in Figure 5d, the battery provides an initial reversible capacity of 118.8 mAh g^−1^ at 70 °C. After 500 cycles, a reversible capacity of 99.9 mAh g^−1^ can still be retained, demonstrating superb cycle life at 70 °C. When the temperature increases to 80, 90, and 100 °C, the all‐organic RPB exhibits 87.08%, 77.13%, and 66.28% capacity retention for 500 cycles, respectively, demonstrating a stable high‐temperature battery. To further evaluate the feasibility for practical applications at high temperatures, we used the lab‐made all‐organic RPBs to power the red LED lights and a small fan in a high‐temperature oven. As shown in Figure 5e,f and Movies S1 and S2, Supporting Information, the all‐organic RPBs are placed in an oven, and then the temperature increases from room temperature to 90 °C in 15 min. After the temperature reaches 90 °C, the all‐organic RPBs are able to power both red LED lights and a small fan in the oven, demonstrating great promise for practical application in the high‐temperature energy storage field. Therefore, the cell configuration based on abundant OEMs and potassium resources is promising for developing fast‐charging, high‐temperature, and sustainable energy storage devices.

### 2.6 The Impact of the Concentrated Electrolyte for the All‐Organic RPB

**Figure 6:**
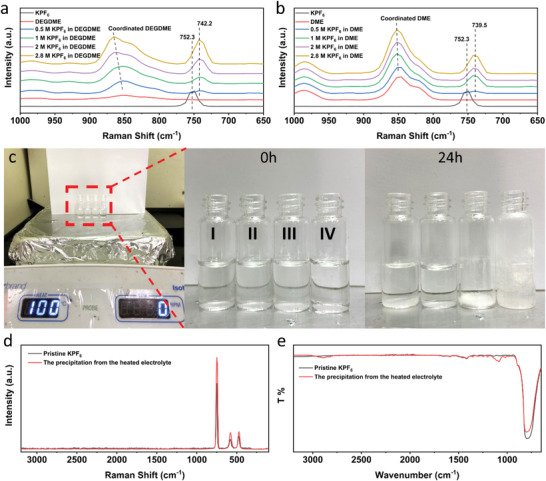
Raman spectra for KPF_6_ and electrolytes with different KPF_6_ concentrations in a) DEGDME and b) DME. c) Different electrolytes heated at 100 °C, 2.8 m KPF_6_ in DEGDME (I), 1 m KPF_6_ in DEGDME (II), 1 m KPF_6_ in DME (III), and 0.8 m KPF_6_ in EC/DEC (IV); d) Raman and e) FTIR spectra of pristine KPF_6_ and the precipitation from the heated 2.8 m KPF_6_‐DEGDME electrolyte.

To gain fundamental insights into the impressive electrochemical performance of the all‐organic RPB, FTIR, Raman spectroscopy, XPS, XRD, and SEM are used to study the electrolyte and SEI structure, as well as the structural evolution of the cycled electrodes at high temperatures. The electrolyte plays an important role in the formation of the stable SEI layer in the fast‐charging and high‐temperature RPB. To understand the impact of the concentrated electrolyte on the electrochemical performance, FTIR and Raman were employed to investigate the chemical properties of various electrolytes. As shown in **Figure** **6**a,b, the P–F bending peak of KPF_6_ at ≈752.3 cm^−1^ undergoes a downshift to 742.2 cm^−1^ when dissolved in the DEGDME solvent, which is smaller than that of KPF_6_ in the DME solvent (739.5 cm^−1^). This demonstrates a stronger K^+^ and PF_6_^−^ interaction in the KPF_6_− DEGDME electrolytes. With increased KPF_6_ concentration up to 2.8 m, the free DEGDME (C—O—C stretching vibration band at ≈850 cm^−1^) shows a more obvious upshift than that in DME‐based electrolytes, indicating the strong K^+^ and DEGDME interaction in the 2.8 m KPF_6_‐DEGDME electrolyte.^[^ ^62^, ^63^, ^64^, ^65^ ^]^ In addition, analogous to the Raman results, the FTIR peaks of C—O—C and C—H groups in DEGDME at ≈1099 cm^−1^ and 843 cm^−1^ show more obvious downshift than that of DME at ≈1104 and 844 cm^−1^ with the increased KPF_6_ concentration (Figure S24c,d, Supporting Information), further confirming a more stable K^+^‐coordinated DEGDME structure in the 2.8 m KPF_6_‐DEGDME electrolyte.^[^ ^66^ ^]^

To further investigate the thermal stability of the concentrated electrolyte, equivalent amount of 2.8 m KPF_6_‐DEGDME, 1 m KPF_6_‐DEGDME, 1 m KPF_6_‐DME, and 0.8 m KPF_6_‐EC/DEC electrolytes were heated at 100 °C in the air. As shown in Figure 6c and Figure S25a–e, Supporting Information, all the electrolytes are clear solutions without any precipitation before the heat treatment. After heating for 1 h, 1 m KPF_6_‐DEGDME and 0.8 m KPF_6_‐EC/DEC electrolytes remain clear. However, the 2.8 m KPF_6_‐DEGDME and 1 m KPF_6_‐DME electrolytes show obvious precipitation. For the 2.8 m KPF_6_‐DEGDME electrolyte, the precipitation starts to form after the temperature reaches 100 °C. To exclude the reaction between the electrolyte and air, the electrolyte was heated to 100 °C in a closed vial and at the Argon atmosphere, but the precipitation still forms. To verify the structure of the precipitation from the heated 2.8 m KPF_6_‐DEGDME electrolyte, the precipitation was collected and dried in a vacuum oven to remove the DEGDME solvent. Raman and FTIR spectroscopies were used to study the structure of the precipitation. As shown in Figure 6d,e, the Raman spectrum of the precipitation is well matched with that of KPF_6_, while the strong FTIR peak at ≈795 cm^−1^ for KPF_6_ presents in the FTIR spectrum for the precipitation, demonstrating that the precipitation is KPF_6_. There are a few small peaks in the FTIR spectrum of the precipitation due to the trace amount of DEGDME solvent (Figure S24c, Supporting Information).^[^ ^66^ ^]^ Hence, the results demonstrate that the extra KPF_6_ forms precipitation at 100 °C. Moreover, the precipitation can be re‐dissolved in DEGDME after the electrolyte cools down to the room temperature. The concentration of 2.8 m KPF_6_‐DEGDME electrolyte is close to saturation at the room temperature, and the solubility of KPF_6_ in DEGDME decreases with the elevated temperature. Extra KPF_6_ forms precipitation at 100 °C. Since the growth of SEI in initial cycles will consume KPF_6_, the precipitation can gradually re‐dissolve into the electrolyte to offset the consumption of KPF_6_ and maintain the electrolyte concentration, which is beneficial for the enhancement of cycling stability of the all‐organic RPB at high temperatures. In addition, the average CE of the all‐organic RPB at 1 A g^−1^ and 80 °C is 99.8% upon long‐term cycling (Figure S26, Supporting Information), indicating continuous consumption of KPF_6_. The KPF_6_ precipitation will compensate for the concentration decrease. Moreover, the 2.8 m KPF_6_‐DEGDME electrolyte with precipitation at 100 °C is still flowable, so it will not compromise the electrochemical performance. In contrast, the volume of 1 m KPF_6_‐DME and 0.8 m KPF_6_‐EC/DEC electrolytes obviously decrease after heating at 100 °C for 3 h (Figure S25, Supporting Information) due to the evaporation of DME and DEC. After 24 h, 1 m KPF_6_‐DME and 0.8 m KPF_6_‐EC/DEC electrolytes become solid‐state, indicating the complete depletion of solvents in these electrolytes. On the contrary, the volume of 2.8 m KPF_6_‐DEGDME and 1 m KPF_6_‐DEGDME electrolytes does not change after heating for 6 h. After 24 h, the volume of 2.8 m KPF_6_‐DEGDME electrolyte is still comparable to that of the pristine electrolyte and higher than that of 1 m KPF_6_‐DEGDME electrolyte, demonstrating the best thermal stability. The TGA of different electrolytes is shown in Figure S25f, Supporting Information. Obviously, the electrolyte of 2.8 m KPF6 in DEGDME exhibits the much higher stability than that of other three electrolytes at the temperature below 100 °C, which is consistent with the result of thermal evaporation. The excellent thermal stability is attributed to the strong interaction between K^+^ and DEGDME in the 2.8 m KPF_6_‐DEGDME electrolyte.

### 2.7 Characterization of the All‐Organic RPB at High Temperatures

**Figure 7:**
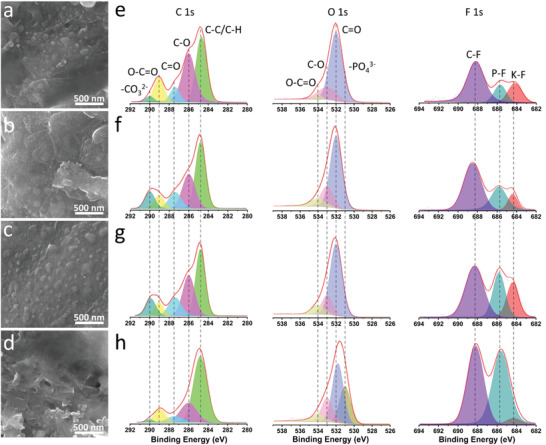
SEM images of the TBPS/NG anode after 20 cycles under the current density of 200 mA g^−1^ at a) 70 °C, b) 80 °C, c) 90 °C, and d) 100 °C; C 1s, O 1s, and F 1s XPS spectra of the TBPS/NG anode after 20 cycles under the current density of 200 mA g^−1^ at e) 70 °C, f) 80 °C, g) 90 °C, and h) 100 °C.

In addition to the electrolyte thermal stability, the SEI and electrode structures at high temperatures were also studied. **Figure** **7**a–d and Figures S27–S30, Supporting Information, show the morphology of the TBPS/NG anode in the all‐organic RPB after 20 cycles at different temperatures. The pristine TBPS/NG anode displays a smooth surface (Figure S12, Supporting Information). At 70 °C, the TBPS/NG particles retain the micro‐sized structure, and the surface is smooth without any cracks. Similar morphologies are also observed at 80 and 90 °C, demonstrating the stable TBPS/NG composite structure and the formation of the stable SEI layer at high temperatures. At 100 °C, the TBPS/NG particles remain as micro‐sized structures and do not show obvious cracks, but the surface becomes rough, and small holes are generated, indicating a less stable composite structure and SEI layer at 100 °C. It matches with the electrochemical performance at high temperatures that the cycling stability of the all‐organic RPB at 100 °C is worse than that at 70–90 °C. Moreover, the morphology of the TBPS anode without NG at high temperatures is also measured as a reference. As shown in Figures S31–S34, Supporting Information, TBPS particles are fully smashed at temperatures above 70 °C, and obvious cracks are formed on the surface of the anode due to the large volume change during the potassiation/de‐potassiation process at high temperatures.

The crystalline structure change of TBPS in the cycled TBPS/NG anode at different temperatures is investigated by XRD (Figure S35, Supporting Information). The sharp peak at ≈18° is ascribed to the polytetrafluoroethylene (PTFE) binder, which is overlapped with one of the XRD peaks for TBPS.^[^ ^41^ ^]^ Compared with the TBPS/NG powder, XRD peaks in the range from 30° to 40° are weakened in the pristine TBPS/NG anode but become stronger after cycling at different temperatures. The peak at 37.5° disappears after cycling at high temperatures, but the peaks at 24.7°, 27.9°, 31.8°, 34.2° and 36.6° remain after cycling at 70–100 °C, indicating that the crystalline structure changes after cycling and the newly formed crystalline structure shows high stability at high temperatures.^[^ ^67^ ^]^ The peaks at 28.89°, 30.9°, 35.29°, and 41.78° are assigned to KF, K_2_CO_3_, carbon phosphorus fluoride (C_12_PF_5.5_), and potassium oxide, which are the key components of the SEI layer. However, the intensity of these peaks at 100 °C is much lower than that at other temperatures due to the instability of the SEI layer at such a high temperature. This is consistent with the electrochemical performance of the all‐organic RPB at high temperatures.

XPS was employed to further investigate the SEI structure at high temperatures (Figure 7e–h). Analogous to the XPS results obtained at room temperature, the pronounced C 1s peak at 290 eV and F 1s peak at ≈684.3 eV are shown after cycling at 200 mA g^−1^ and 70 °C for 20 cycles, demonstrating the presence of K_2_CO_3_ and KF in the SEI layer. These two peaks maintain a high intensity at 80 and 90 °C but are dramatically weakened at 100 °C, indicating the reduced K_2_CO_3_ and KF content in the SEI layer at 100 °C. In addition, the intensity of F 1s peaks at ≈685.8 and ≈688.2 eV, and P 2p peaks at ≈134.1 and ≈138.3 eV is dramatically enhanced at 100 °C (Figure 7h and Figure S36, Supporting Information), demonstrating that the content of organic fluoride and phosphate in the SEI is remarkably increased at 100 °C due to the continuous parasitic reactions at the anode/electrolyte interphase. A thick fluoro‐organic layer in the SEI will hinder the electronic and ionic conductivity of the anodes and result in worse electrochemical performance at 100 °C. This result further proves that K_2_CO_3_ and KF are key components in the SEI to improve the stability of the TBPS/NG anode at high temperatures.

### 2.8 Characterization of the Cycled PANI/NG Cathode at High Temperature

The structure stability of the PANI/NG cathode in the all‐organic RPB is also very important for the electrochemical performance, so we used SEM and XPS to study the morphology change and surface structure of the cycled PANI/NG cathode at high temperatures. In the pristine PANI/NG cathode, the surface of PANI/NG particles are smooth and the PANI is fully covered by NG (Figure S37, Supporting Information). After cycled at 70 °C, the NG on the surface of the particles is wrinkled, and the surface becomes rougher as the temperature rises to 100 °C (Figures S38–S41, Supporting Information). However, there are no obvious cracks on the surface of cycled PANI/NG particles even at 100 °C. The comparison between the morphologies of PANI/NG cathode and TBPS/NG anode after cycling at 100 °C proves that the PANI/NG cathode shows much better structural integrity than that of the TBPS/NG anode, which suggests that the structural deterioration of the TBPS/NG anode is the main reason for the capacity decay of the all‐organic RPB at 100 °C. To verify this conclusion, XPS was further employed to investigate the cathode electrolyte interphase (CEI) (Figure S42, Supporting Information) formation at high temperatures.^[^ ^26^ ^]^ The peak at 531 eV in O 1s XPS spectra is assigned to —PO_4_^3−^, indicating the existence of phosphate in the CEI layer. The intensity of the —PO_4_^3−^ peak does not change from 70 to 100 °C, demonstrating high stability of the CEI layer at high temperatures. The intensity of peaks at ≈688 and ≈685 eV in F 1s XPS spectra dramatically increases at 90 and 100 °C, revealing the thickness of organic fluorides and KF increases over 90 °C. The results demonstrate that the CEI is composed of phosphate, organic fluorides, and KF, which exhibit superior stability even at a high temperature of 100 °C.

To further improve the stability of all‐organic K batteries, especially at high temperature, there are several approaches: 1) using the artificial SEI or CEI consisting of KF and K_2_CO_3_ to protect the anode and cathode, alleviating the consumption of electrolyte by continuous SEI/CEI formation during repeated charge/discharge process; 2) optimizing the electrolyte to achieve stable and robust KF‐riched SEI or CEI;^[^ ^48^, ^49^, ^50^, ^51^, ^52^, ^53^, ^54^, ^55^, ^56^, ^57^, ^58^, ^59^, ^60^, ^61^, ^62^, ^63^, ^64^, ^65^, ^66^, ^67^, ^68^, ^69^, ^70^, ^71^, ^72^ ^]^ 3) employing multi‐dimensional conductive additives to accommodate the volume changes of anode and cathode materials and form stable SEI/CEI during repeated potassiation/de‐potassiation process.^[^ ^73^, ^74^, ^75^, ^76^, ^77^ ^]^

## 3 Conclusion

In summary, this work demonstrates the feasibility of employing abundant and sustainable OEMs and potassium resources to achieve fast‐charging and high‐temperature batteries. An organic anode material, TBPS, was synthesized to couple with PANI for the all‐organic RPB at extreme conditions. NG and a 2.8 m KPF_6_‐DEGDME electrolyte are used to mitigate the dissolution of OEMs, enhance the conductivity of organic electrodes, accommodate the large volume change, generate stable electrode/electrolyte interphases, and thus improving the electrochemical performance of the all‐organic RPB, especially under extreme conditions. The TBPS/NG anode delivers excellent electrochemical performance, in terms of high specific capacity (225.5 mAh g^−1^ at 50 mA g^−1^), fast‐charging capability (up to 10 A g^−1^), and long cycling stability (5000 cycles at 5 A g^−1^). After coupling with the PANI/NG cathode, an all‐organic RPB exhibits ultralong cycle life of 50000 cycles at 5 A g^−1^ and room temperature. The all‐organic RPB with high mass loading of 6.5 mg cm^−2^ also shows exceptional performance at high temperatures that stable cycle life of 500 cycles at 1 A g^−1^ and 70–100 °C can be achieved. The superior electrochemical performance at both room temperature and high temperature is attributed to the stable organic anode/cathode materials (TBPS and PANI), highly conductive and robust NG, as well as the formation of a K_2_CO_3_‐ and KF‐rich SEI. Therefore, our findings open up an opportunity for the design and fabrication of fast‐charging, high‐temperature, and sustainable batteries.

## 4 Experimental Section

### Materials

Tetrahydroxy‐1,4‐benzoquinone hydrate (TCI, >98%), PANI (Alfa Aesar, >98%), N‐doped graphene (ACS Material) were used as received. The TBPS was prepared as follows: tetrahydroxy‐1,4‐benzoquinone hydrate was dispersed in ethanol with potassium hydroxide in 5% excess. The solution was stirred at room temperature for 48 h, then the solution was filtered to collect the precipitate. The precipitate was washed with ethanol and dried in the vacuum oven at 90 °C overnight. 60 mg of the obtained TBPS powder was loaded in a 5 mL vial together with 30 mg of N‐doped graphene, then ball milling at 2000 rpm for 10 min was performed to prepare the TBPS/NG powder using Spex SamplePrep 5120 Mini Mixer/Mill Grinder. Similarly, the PANI powder was stored in a vial together with N‐doped graphene with the mass ratio of 2:1, then ball milling at 2000 rpm for 10 min was performed to prepare the PANI/NG powder.

### Characterizations

X‐ray diffraction (XRD) pattern was recorded by Rigaku MiniFlex using CuK*α* radiation; Fourier transform infrared spectroscopy (FTIR) was recorded by Agilent Cary 630 FTIR Spectrometer; Nuclear magnetic resonance (NMR) was recorded by Bruker Ascend 400; The morphologies of electrode materials were observed by SEM (JEOL JSM‐IT500HR) and TEM (JEOL JEM‐1400F); Raman spectroscopy was recorded by Horiba XploRA PLUS Raman microscope with a 532 nm laser. XPS measurements were carried out at a PHI 5000 VersaProbe II system (Physical Electronics) spectrometer, which is equipped with a hemispherical analyzer. The spectrometer is attached to the Ar glovebox and sample transfer was directly through it to avoid any contact of the samples with air and moisture. Monochromatic Al‐K*α* excitation (*hν* = 1486.6 eV) was used at power of 25 W, additionally applying a low‐energy electron charge neutralizer. The high‐resolution spectrum of each element was collected with a pass energy of 23.25 eV in an analysis area of 100×100 µm. The binding energy scale was corrected based on the C1s peak from contaminations (C—C at 284.8 eV) or from the amorphous carbon (around 284.0 eV) as internal binding energy standard.

### Electrochemical Measurements

The TBPS/NG powder was mixed with carbon black and sodium alginate binder to form a slurry at the weight ratio of 80:10:10, in which the weight fractions of TBPS and NG are 53.33% and 26.67%, respectively. The electrode was prepared by casting the slurry onto Al foil using a doctor blade and dried in a vacuum oven at 90 °C overnight to prepare the TBPS/NG anode. The slurry coated on Al foil was punched into circular electrodes with a mass loading of ≈1.8 mg cm^−2^ based on TBPS. The PANI/NG powder was mixed with carbon black and sodium alginate binder to form a slurry at the weight ratio of 80:10:10, in which the weight fractions of PANI and NG are 53.33% and 26.67%, respectively. The electrode was prepared by casting the slurry onto Al foil using a doctor blade and dried in a vacuum oven at 90 °C overnight to prepare the PANI/NG cathode. The slurry coated on Al foil was punched into circular electrodes with a mass loading of ≈2.7 mg cm^−2^ based on PANI. For the preparation of electrodes with high mass loading (6.5 mg cm^−2^, based on the mass of TBPS, and 9.7 mg cm^−2^, based on PANI), the TBPS/NG or PANI/NG powder was mixed with carbon black and PTFE binder at the weight ratio of 80:10:10, a thick film was obtained directly after grinding for 20 min. The thick film was cut into small pieces, and each piece was pressed together with a stainless‐steel mesh. The anode or cathode with high mass loading was obtained after drying in a vacuum oven at 90 °C overnight.

Coin cells (2032) for rechargeable potassium batteries were assembled using K metal as the counter electrode, a 2.8 m KPF_6_ in DEGDME electrolyte and glass fiber (Whatman) as the separator. For the half cell, the current densities were set based on the mass of TBPS or PANI. The specific capacities of the anode or cathode in half cell (C, mAh g^−1^) were calculated using the following equation:

(3) $$C=\frac{I\times t}{m\times 3.6}$$

where *I* is the current based on the mass of TBPS for anode or PANI for cathode (mA); *t* is the discharge time of the electrodes (s); *m* is the mass loading of active components (TBPS or PANI) or the total mass of electrodes including these active components and N‐doped graphene (TBPS/NG or PANI/NG) (mg).

To assemble the all‐organic battery, TBPS/NG anode and PANI/NG cathode were activated under the current density of 100 mA g^−1^ based on the mass of TBPS or PANI for ten cycles using the half‐cell system. After cycling, coin cells were opened in glovebox and re‐assemble the all‐organic battery using TBPS/NG as the anode and PANI/NG as the cathode. Electrochemical performance was tested using Landt battery test system. For the all‐organic battery, the current densities were set based on the mass of TBPS. The specific capacities of the all‐organic battery (*C*, mAh g^−1^) were also calculated using the Equation (1), where *I* is the current based on the mass of TBPS (mA); *t* is the discharge time of the all‐organic battery (s); *m* is the mass loading of TBPS or the total mass of TBPS/NG (mg). Cyclic voltammograms were recorded using Gamry Reference 1010E Potentiostat/Galvanostat/ZRA with a scan rate of 0.1–1 mV s^−1^. Impedance analysis was also performed by Gamry Reference 1010E Potentiostat/Galvanostat/ZRA. To do the XRD test before and after cycling at high temperatures, four all‐organic batteries were assembled. After running for 20 cycles at 70, 80, 90, and 100 °C under the current density of 200 mA g^−1^ and kept at 0.05 V for 5 h, the coin cells were opened in the glovebox. The four electrodes were washed by DEGDME and dried in a vacuum oven at 90 °C overnight. Then, the four cycled electrodes and pristine electrode were directly tested by Rigaku MiniFlex.

### Statistical Analysis

Each experiment about electrochemical performance was repeated three times wherever not mentioned. All the data are represented as mean and visualized by the Origin Lab software.

## Conflict of Interest

The authors declare no conflict of interest.

## Supporting information

Supporting Information

Supplemental Movie 2

Supplemental Movie 2

## Data Availability

The data that support the findings of this study are available from the corresponding author upon reasonable request.
