# Supplementary material for: A Fast‐Charging and High‐Temperature All‐Organic Rechargeable Potassium Battery
Source: Adv Sci (Weinh). 2022 Oct 31;9(34):2106116. doi: 10.1002/advs.202106116 (PMC9731705; doi:10.1002/advs.202106116)
Supplement: Supplementary file 1 — Supporting Information [file ADVS-9-2106116-s001.pdf]

ADVANCED SCIENCE
Open Access

Supporting Information

for *Adv. Sci.*, DOI 10.1002/advs.202106116

A Fast-Charging and High-Temperature All-Organic Rechargeable Potassium Battery

*Kaiqiang Qin, Kathryn Holguin, Jinghao Huang, Motahareh Mohammadiroudbari, Fu Chen, Zhenzhen Yang, Gui-Liang Xu and Chao Luo**

Supporting Information

A fast-charging and high-temperature all-organic rechargeable potassium battery

Kaiqiang Qin,[1] Kathryn Holguin,[1] Jinghao Huang,[1] Motahareh Mohammadiroudbari,[1] Fu Chen,[2] Zhenzhen Yang,[3] Guiliang Xu,[3] Chao Luo*[1,4]

[1]Department of Chemistry and Biochemistry, George Mason University, Fairfax, VA, 22030, USA

[2]Department of Chemistry and Biochemistry, University of Maryland, College Park, MD, 20742, USA

[3]Chemical Sciences and Engineering Division, Argonne National Laboratory, Lemont, IL, 60439, USA

[4]Quantum Science & Engineering Center, George Mason University, Fairfax, VA, 22030, USA

*Corresponding author: cluo@gmu.edu

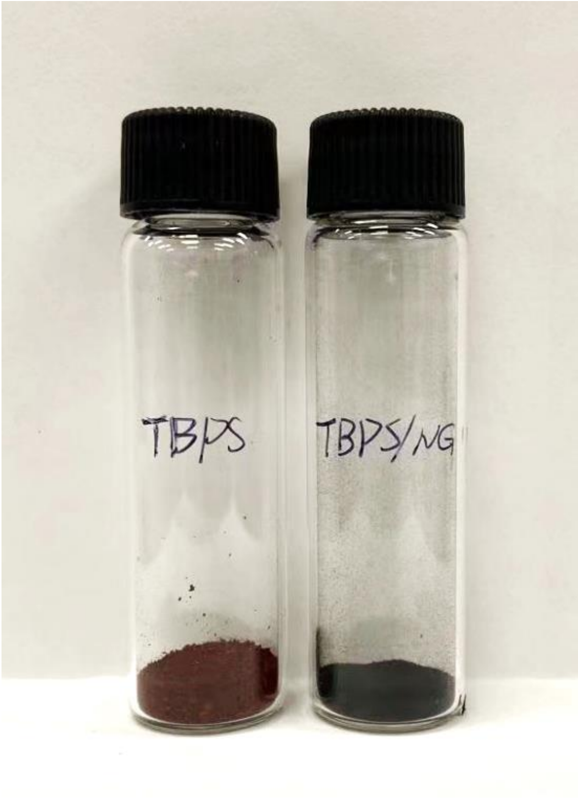

Figure S1. Digital photos of TBPS and TBPS/NG powders.

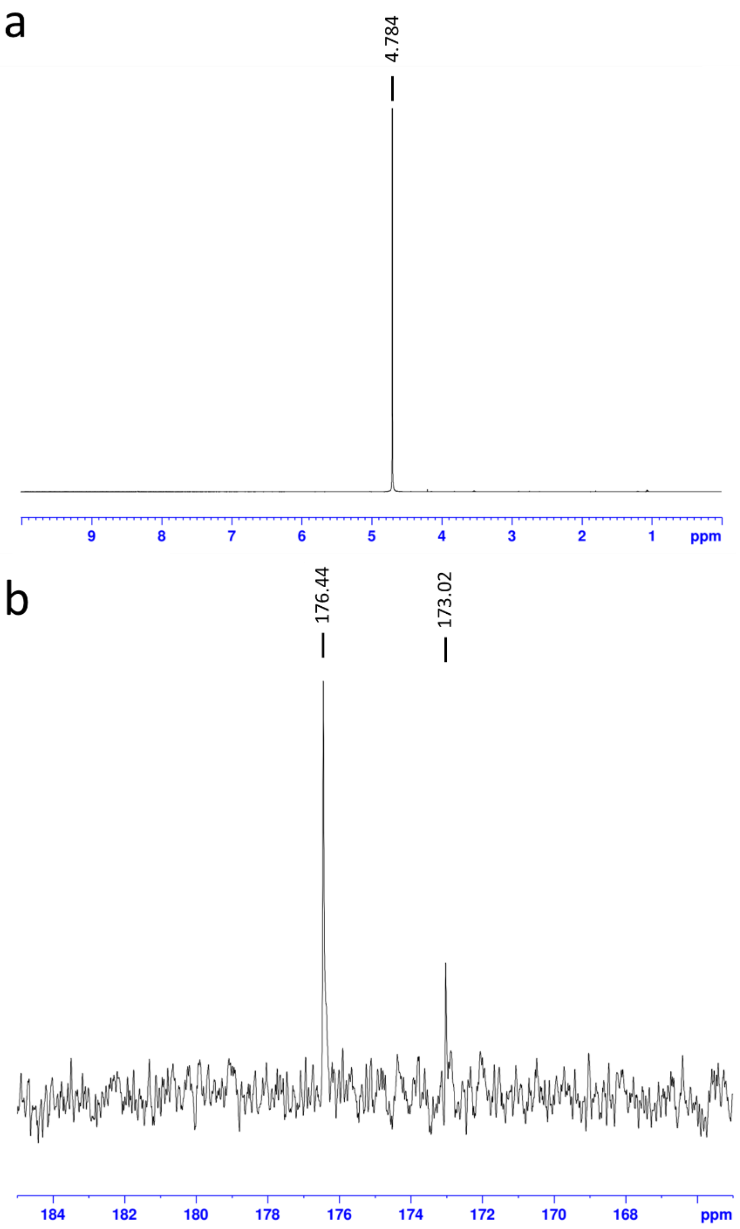

Figure S2. (a) ^1H NMR and (b) ^{13}C NMR for the TBPS compound with D_2O as the solvent.

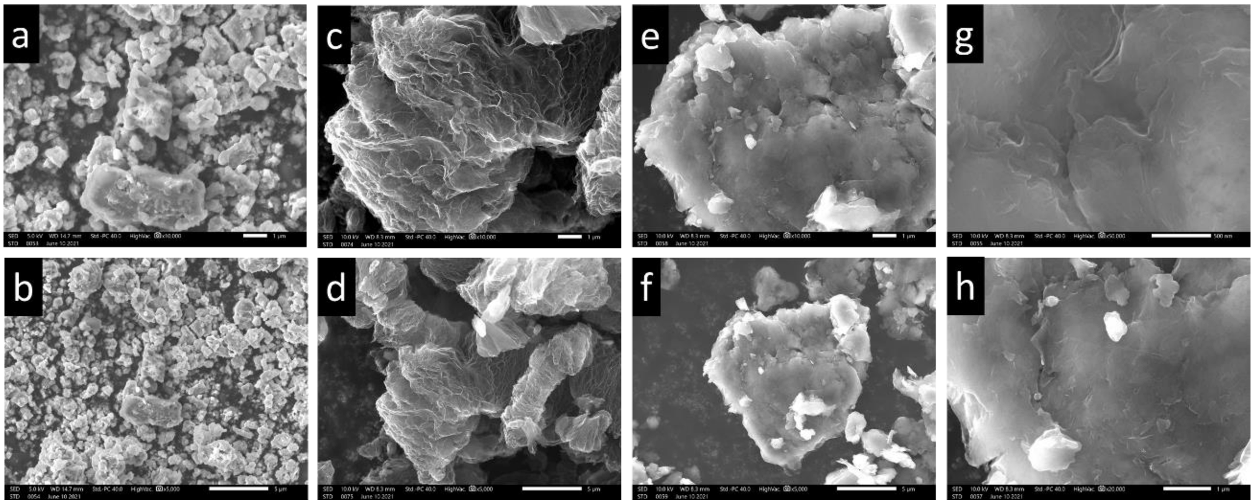

Figure S3. SEM images of (a, b) TBPS, (c, d) N-doped graphene, and (e, f) TBPS/NG powders. (g, h) The high magnification SEM of TBPS/NG powders.

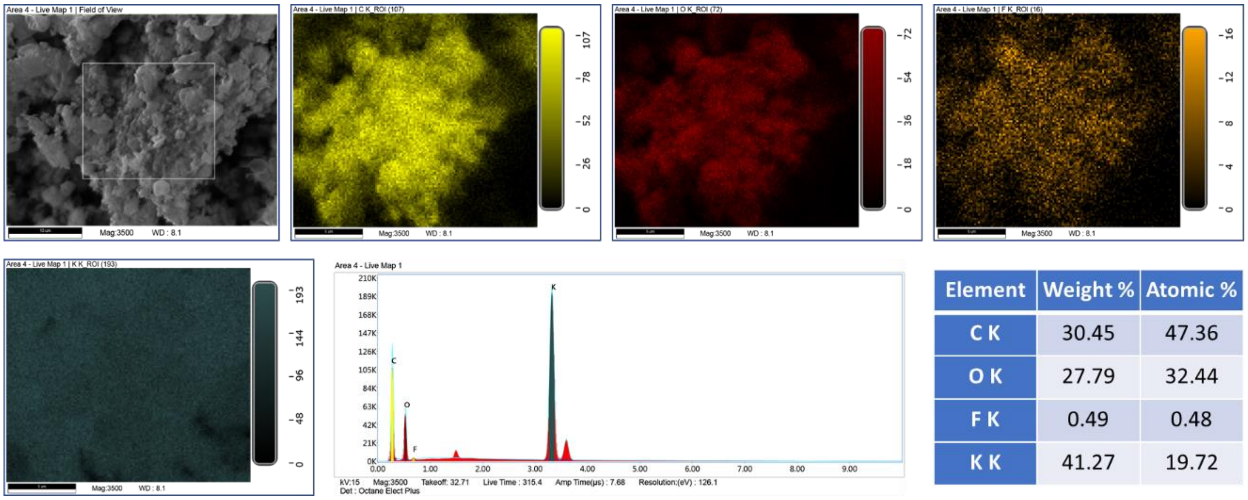

Element	Weight %	Atomic %
C K	30.45	47.36
O K	27.79	32.44
F K	0.49	0.48
K K	41.27	19.72

Figure S4. SEM elemental mappings and EDX of the TBPS powder.

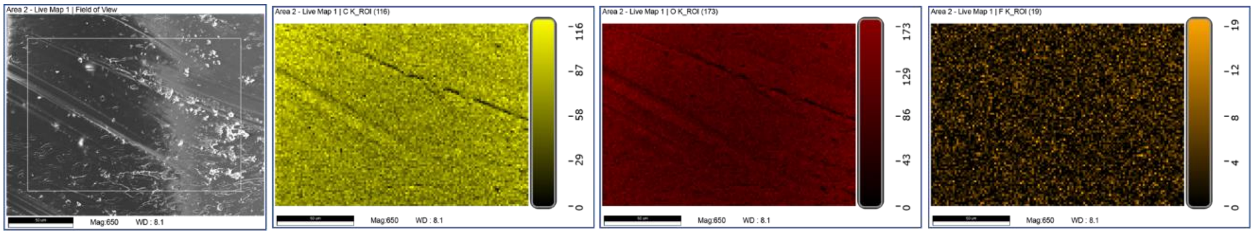

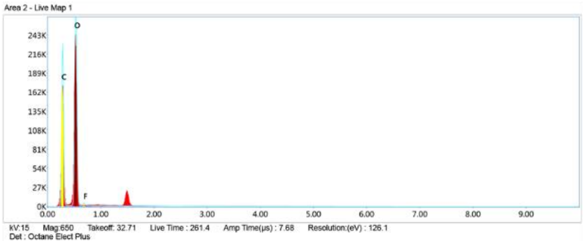

Element	Weight %	Atomic %
C K	50.59	57.76
O K	48.67	41.71
F K	0.74	0.53

Figure S5. SEM elemental mappings and EDX of tetrahydroxy-1,4-benzoquinone hydrate precursor.

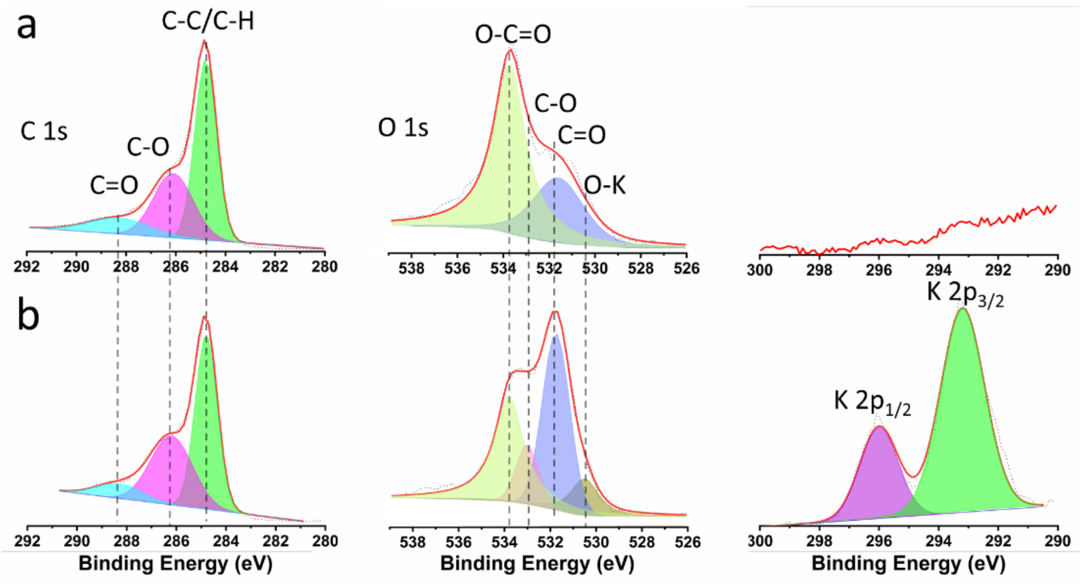

Figure S6. XPS C 1s, O 1s, and K 2p spectra of (a) N-doped graphene and (b) TBPS/NG powders.

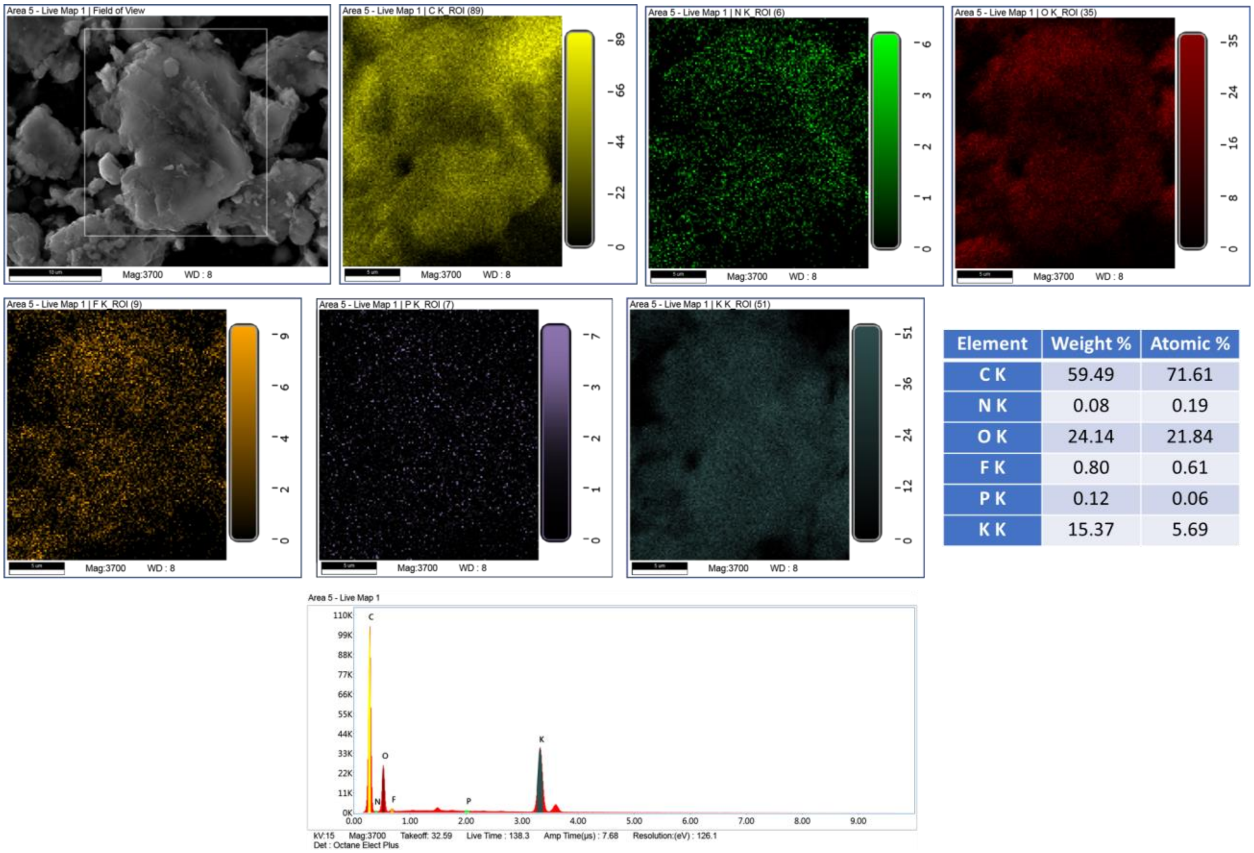

Element	Weight %	Atomic %
C K	59.49	71.61
N K	0.08	0.19
O K	24.14	21.84
F K	0.80	0.61
P K	0.12	0.06
K K	15.37	5.69

Figure S7. SEM elemental mappings and EDX of the TBPS/NG powder.

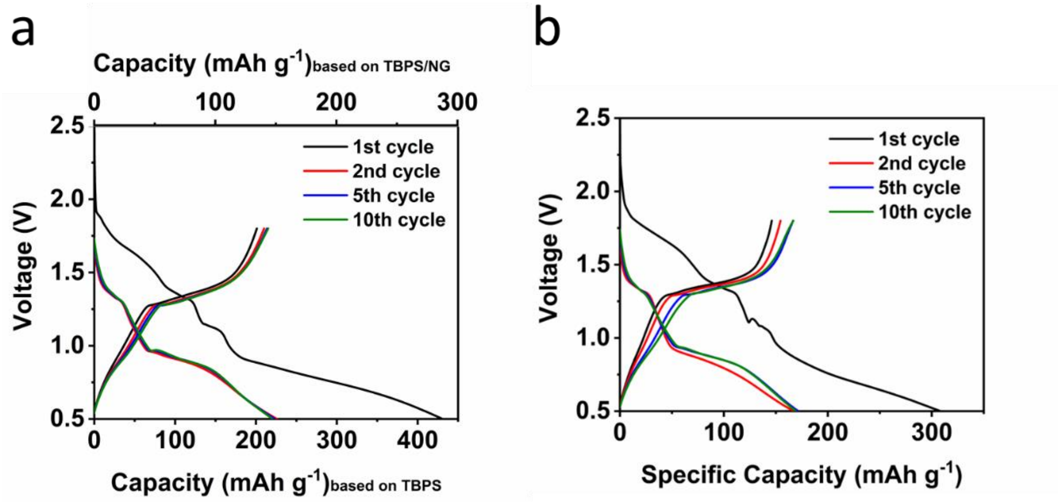

Figure S8. Galvanostatic charge/discharge curves of (a) TBPS/NG and (b) TBPS anodes at 50 mA g^{-1}.

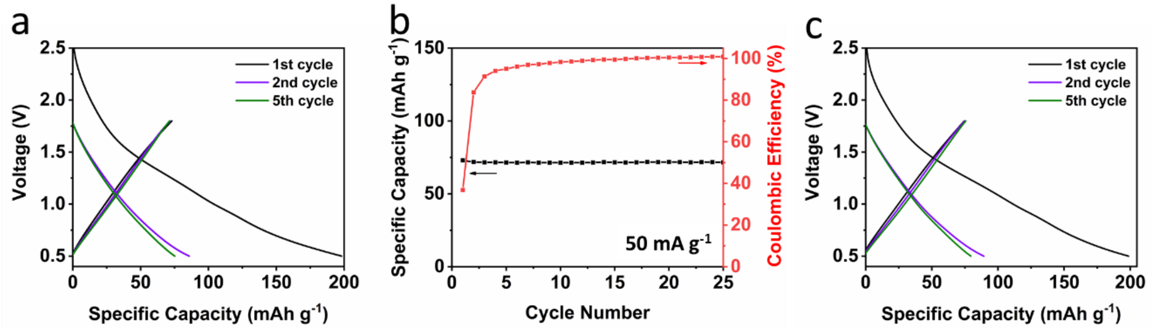

Figure S9. Electrochemical performance of NG electrodes. (a) Galvanostatic charge/discharge curves and (b) cycling stability of NG-I electrode at the current density of 50 mA g^{-1}. (c) Galvanostatic charge/discharge curves of NG-II electrode at the current density of 50 mA g^{-1}.

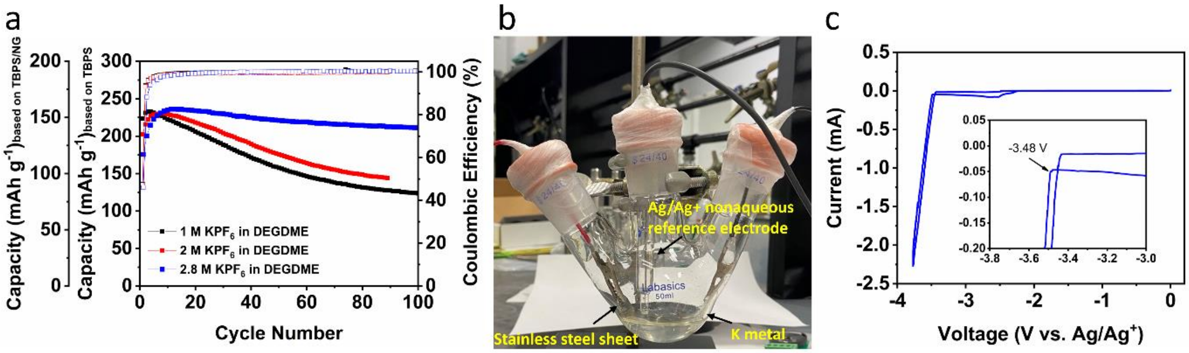

Figure S10. (a) Charge capacities and Coulombic efficiency of TBPS/NG anodes measured at 100 mA g^{-1} using 1M KPF_6 in DEGDME, 2M KPF_6 in DEGDME, and 2.8M KPF_6 in DEGDME electrolytes. (b) The three-electrode system employed for the investigation of redox potential of K in the electrolyte of 2.8M KPF_6 in DEGDME using stainless steel sheet as the working electrode, K metal as the counter electrode and Ag/Ag^+ nonaqueous electrode (filled with 10mM $AgNO_3$ in CH_3CN) as the reference electrode. (c) CV curves of three-electrode system with 2.8M KPF_6 in DEGDME under scan rate of 0.1 mV s^{-1} at voltage range of -3.8-0 V (vs. Ag/Ag).

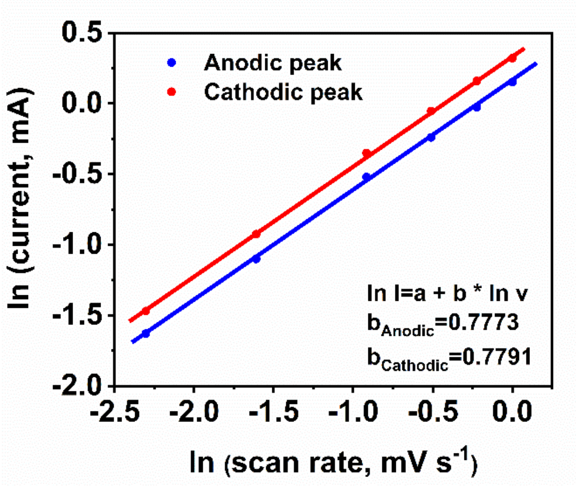

Figure S11. The ln relationship of peak current and scan rate.

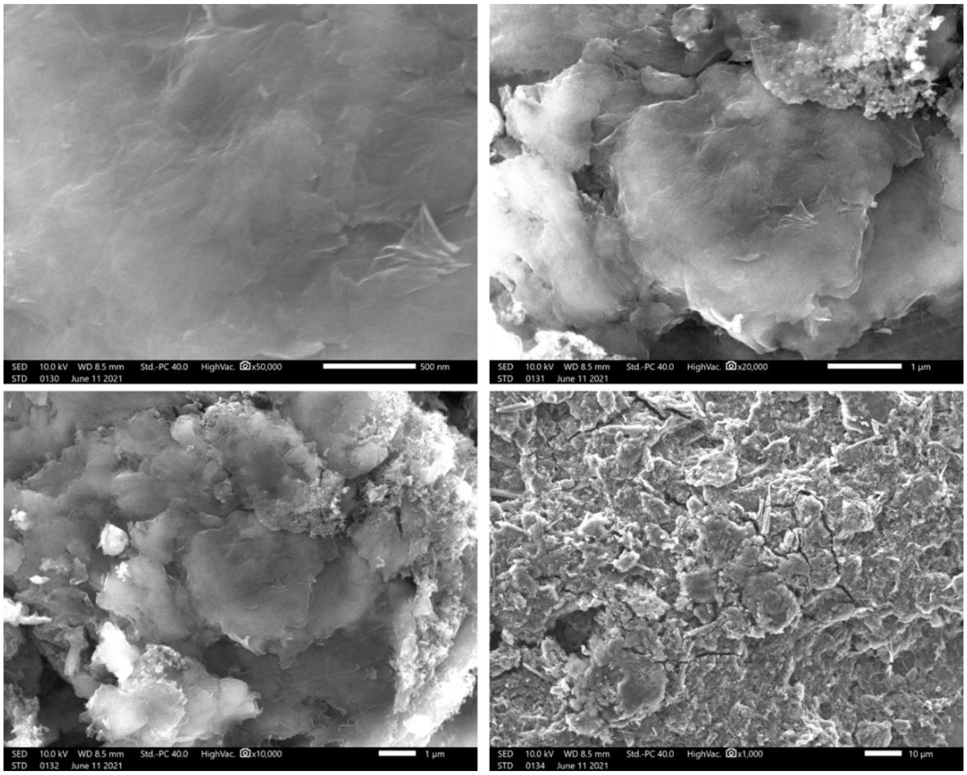

Figure S12. SEM images of the pristine TBPS/NG anode.

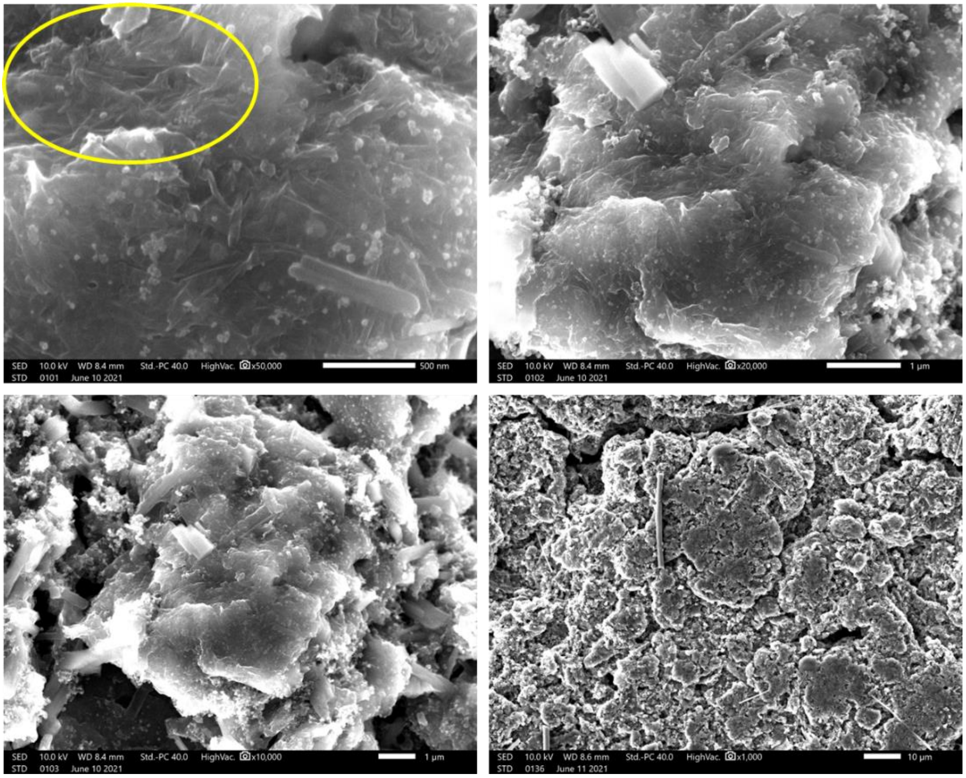

Figure S13. SEM images of the TBPS/NG anode after 20 cycles at the current density of 200 mA g^{-1}.

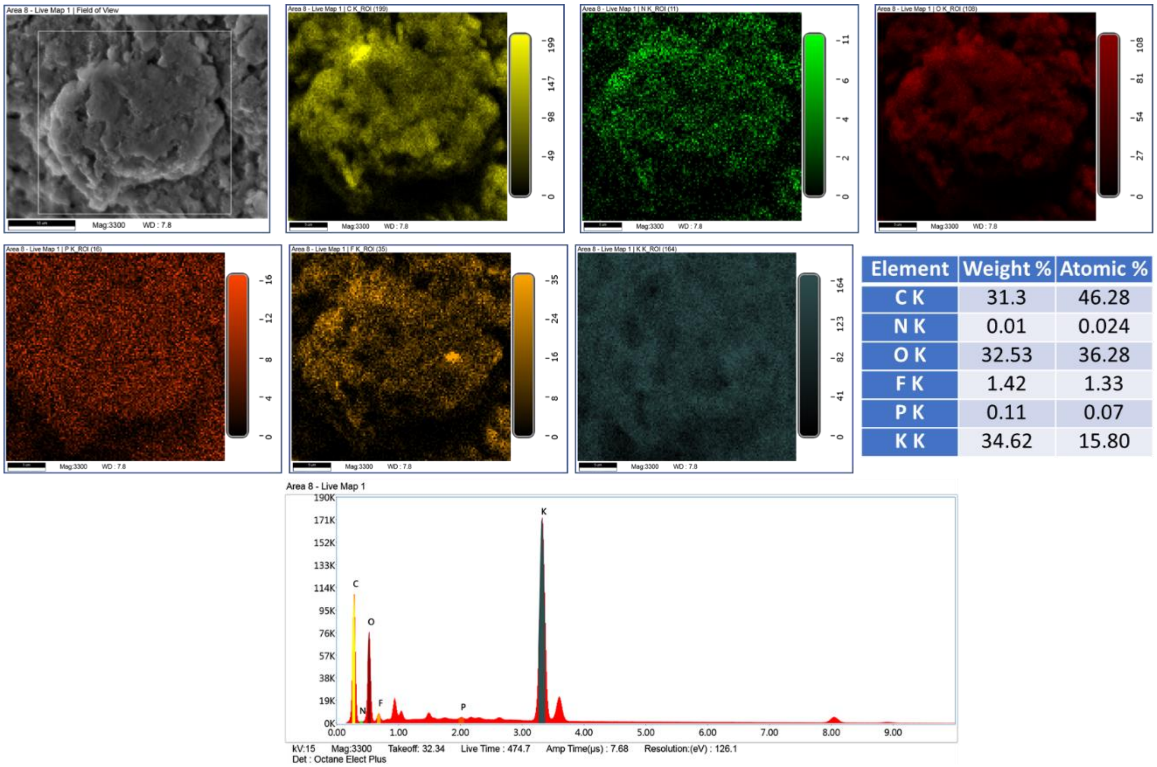

Element	Weight %	Atomic %
C K	31.3	46.28
N K	0.01	0.024
O K	32.53	36.28
F K	1.42	1.33
P K	0.11	0.07
K K	34.62	15.80

Figure S14. SEM elemental mappings and EDX of TBPS/NG anode after 20 cycles at the current density of 200 mA g^{-1}.

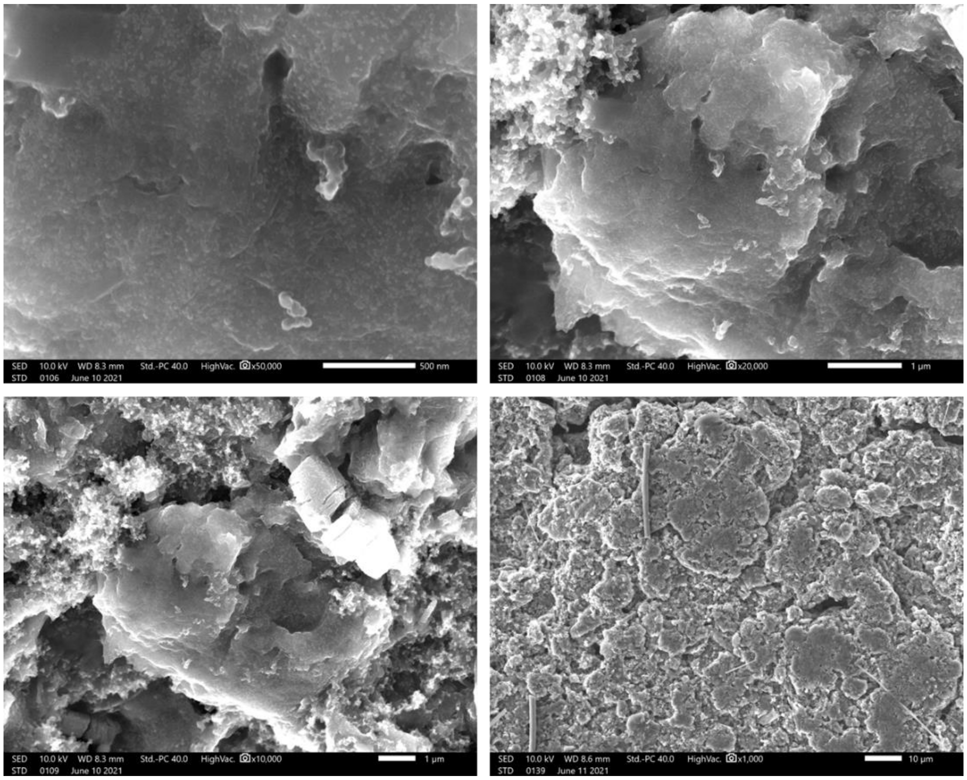

Figure S15. SEM images of TBPS/NG anode after 20 cycles at the current density of 5 A g^{-1}.

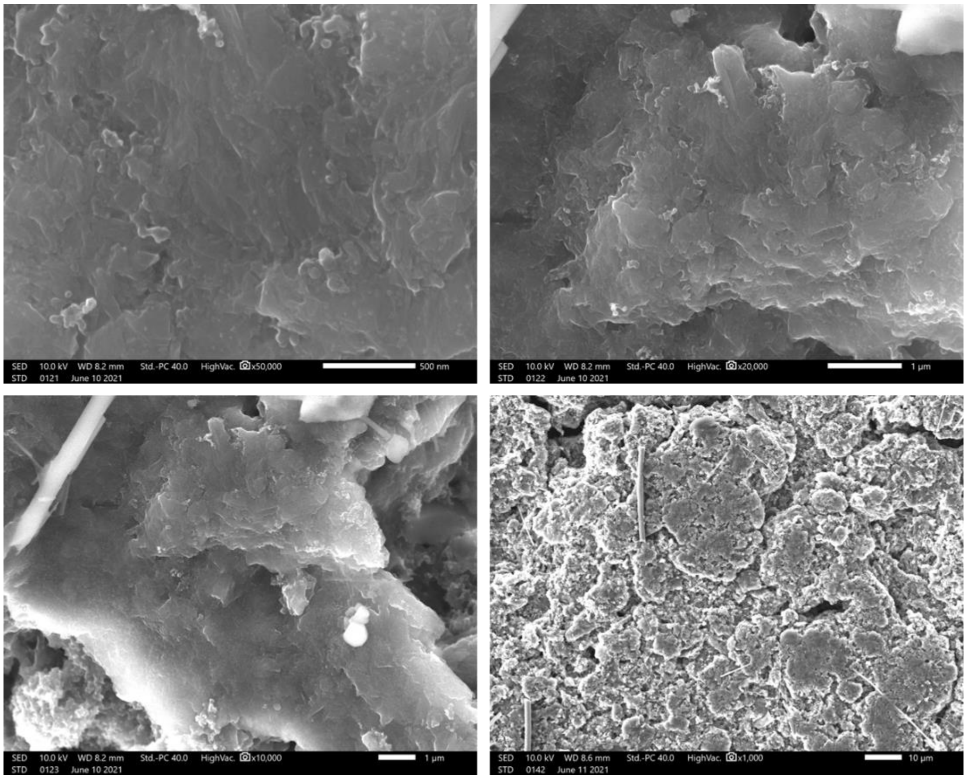

Figure S16. SEM images of TBPS/NG anode after 20 cycles at the current density of 10 A g^{-1}.

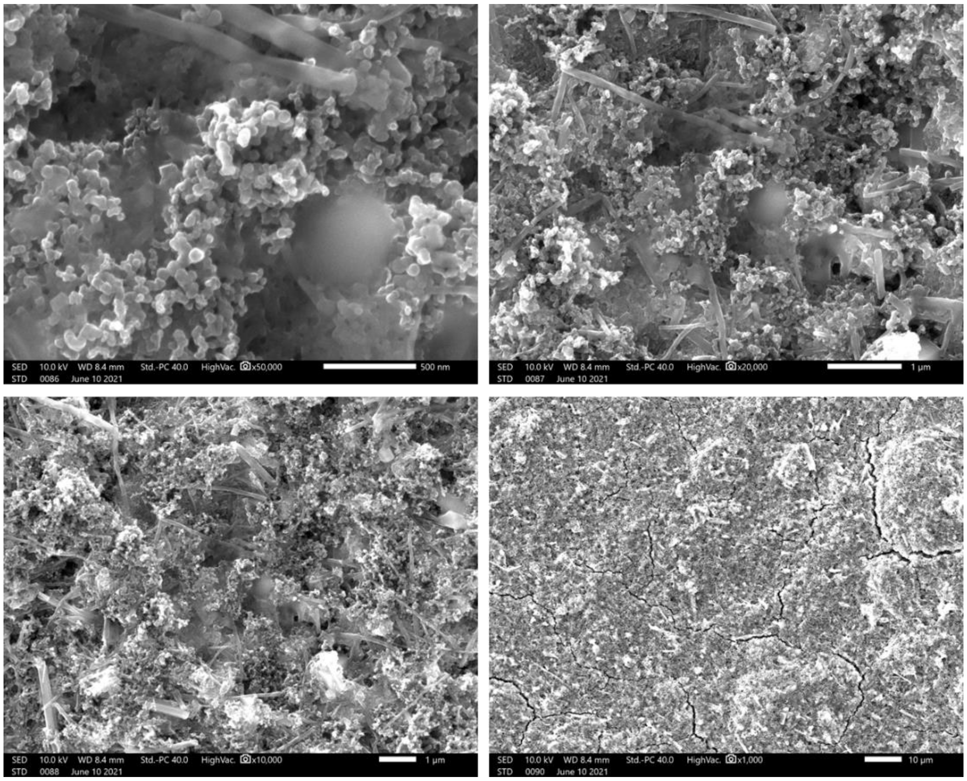

Figure S17. SEM images of the pristine TBPS anode.

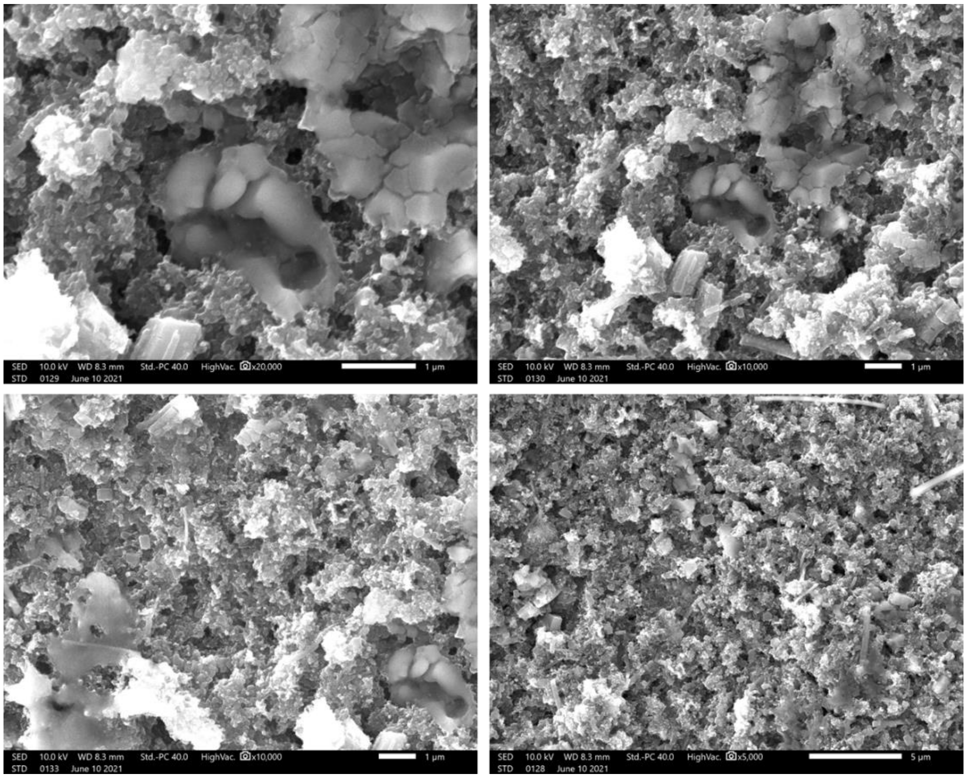

Figure S18. SEM images of TBPS anode after 20 cycles at the current density of 200 mA g^{-1}.

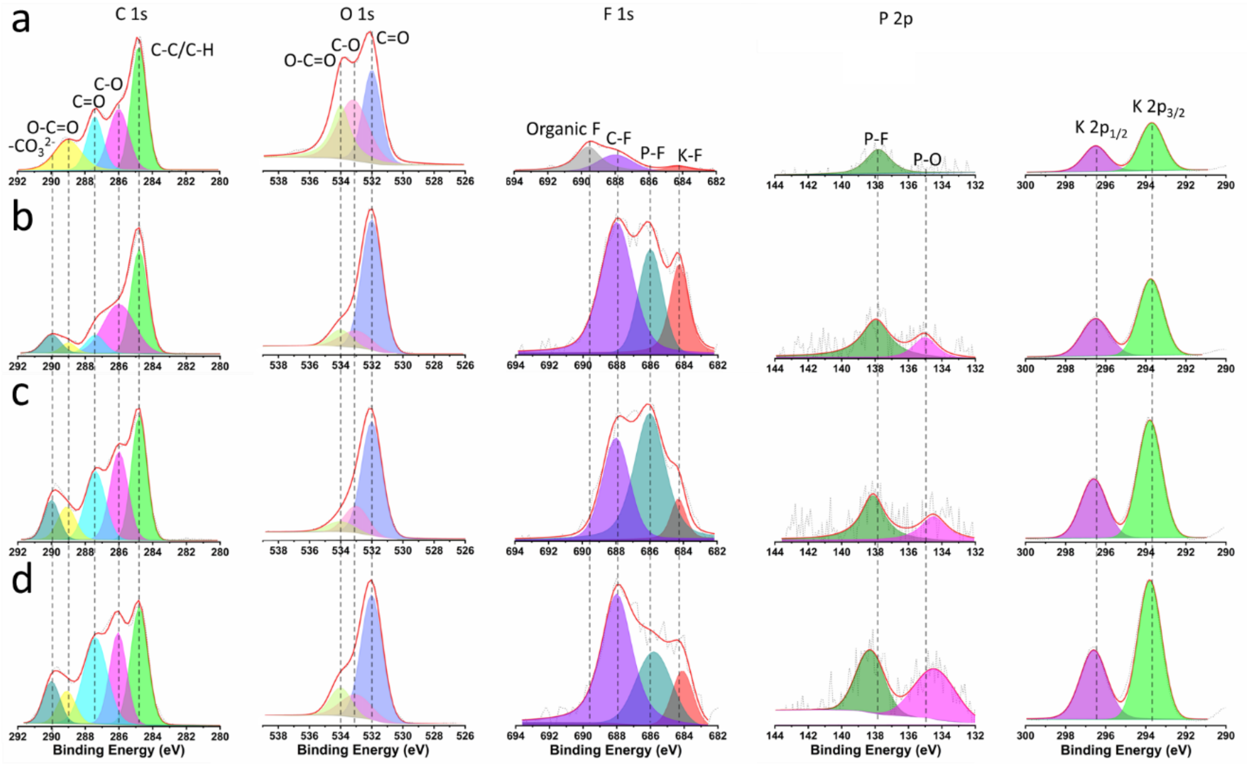

Figure S19. XPS spectra of (a) the pristine TBPS/NG anode, and the TBPS/NG anode after 20 cycles at the current densities of (b) 200 mA g^{-1}, (c) 5 A g^{-1}, and (d) 10 A g^{-1}.

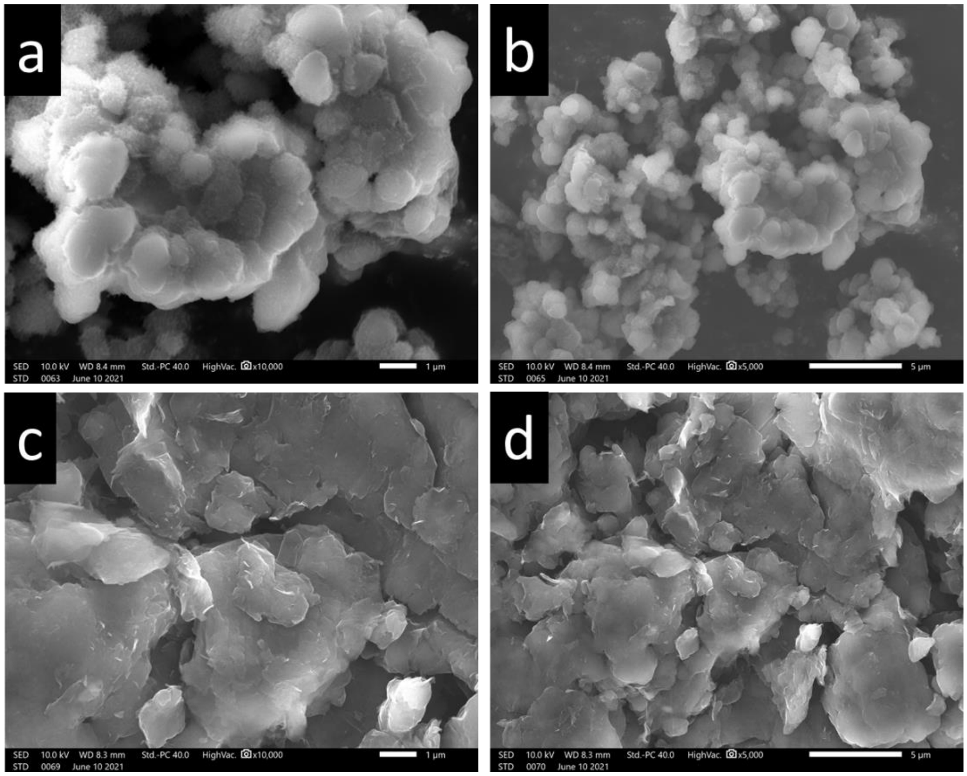

Figure S20. SEM images of (a, b) the pristine PANI powder and (c, d) the PANI/NG composite powder.

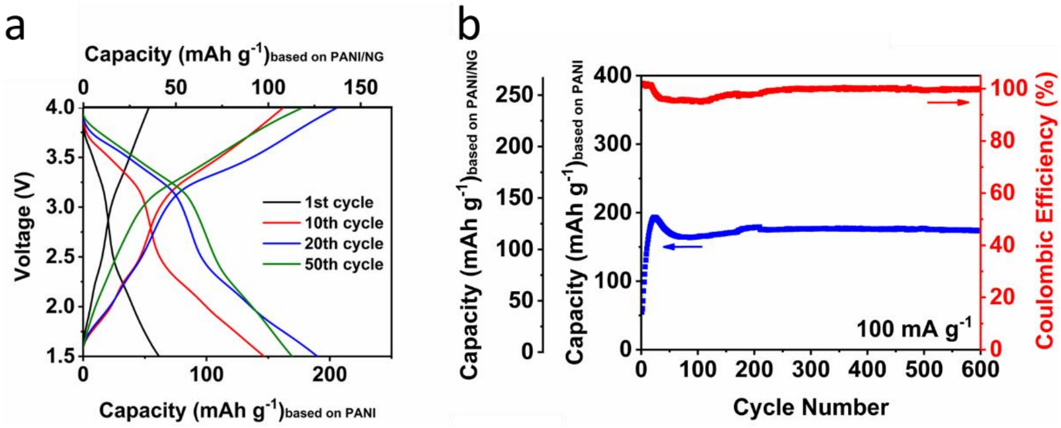

Figure S21. Electrochemical performance of the PANI/NG cathode. (a) Galvanostatic charge/discharge curves and (b) cycling stability at the current density of 100 mA g^{-1}.

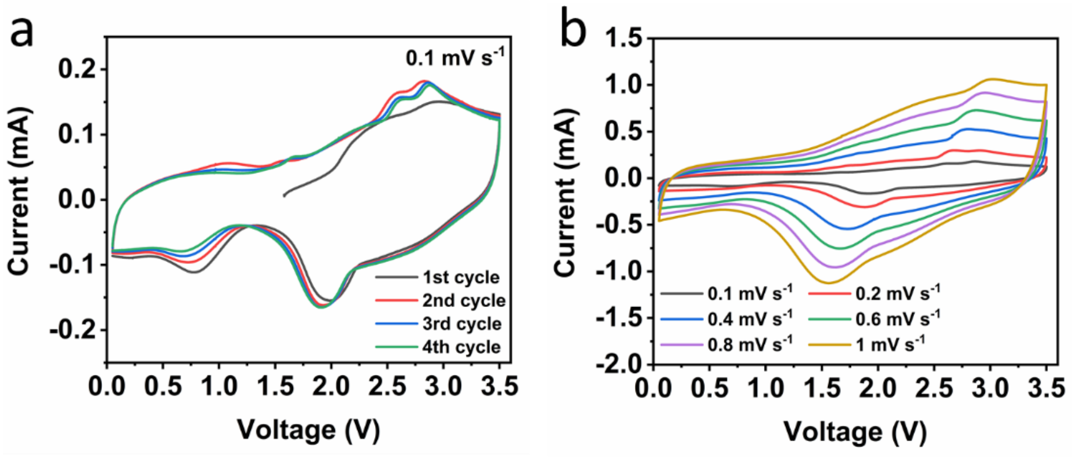

Figure S22. Cyclic voltammograms of the all-organic potassium battery at (a) 0.1 mV s^{-1} and (b) various scan rates.

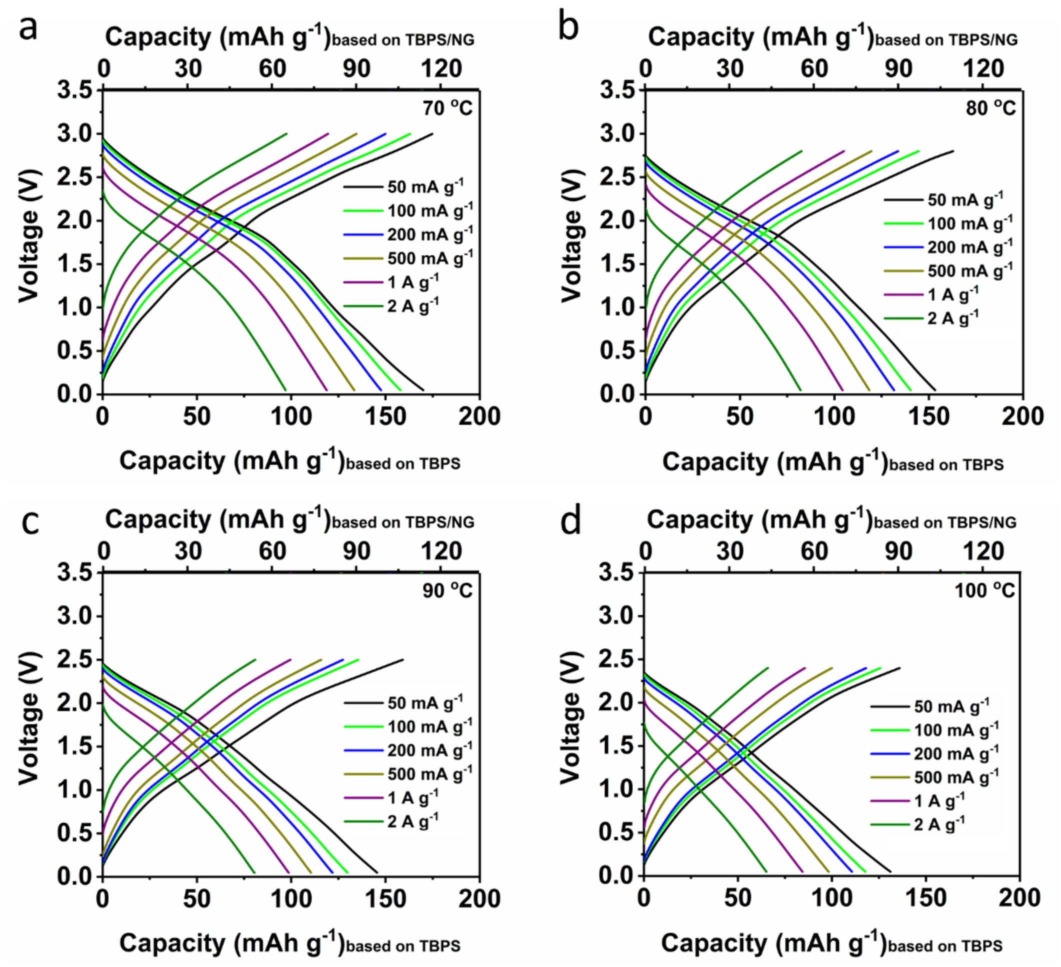

Figure S23. Galvanostatic charge/discharge curves under different current densities at (a) 70°C, (b) 80°C, (c) 90°C, and (d) 100°C.

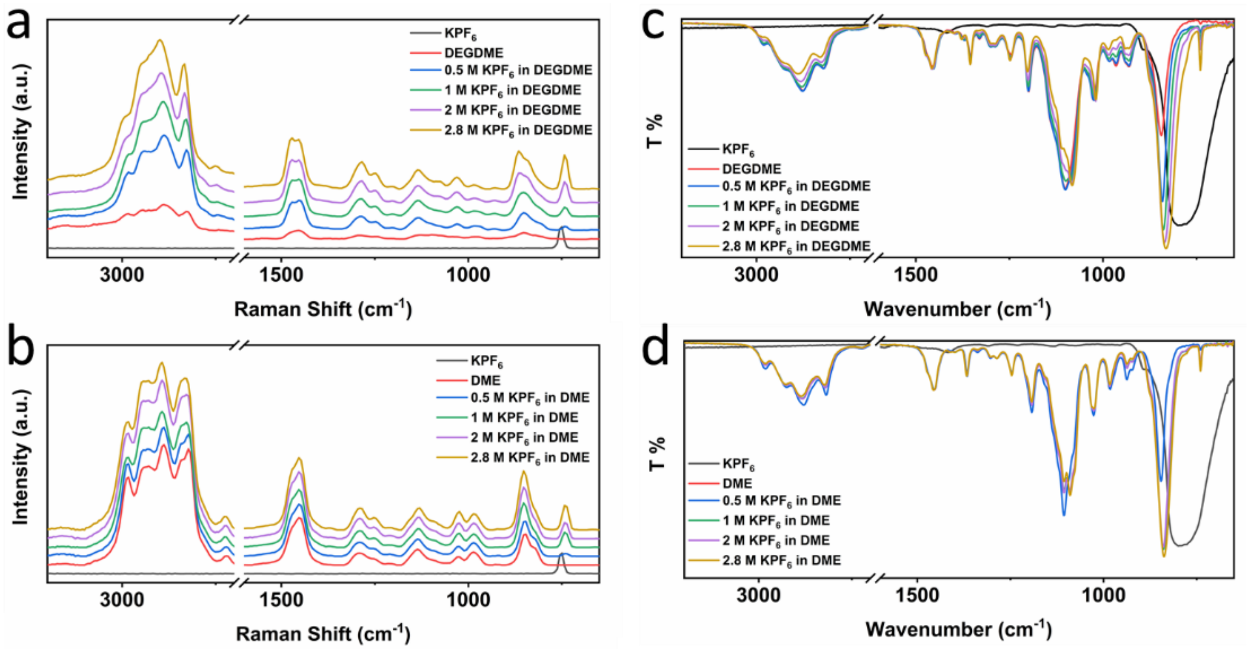

Figure S24. Raman spectra for KPF_6 and electrolytes with different KPF_6 concentrations in (a) DEGDME and (b) DME; FTIR spectra for KPF_6 and electrolytes with different KPF_6 concentrations in (c) DEGDME and (d) DME.

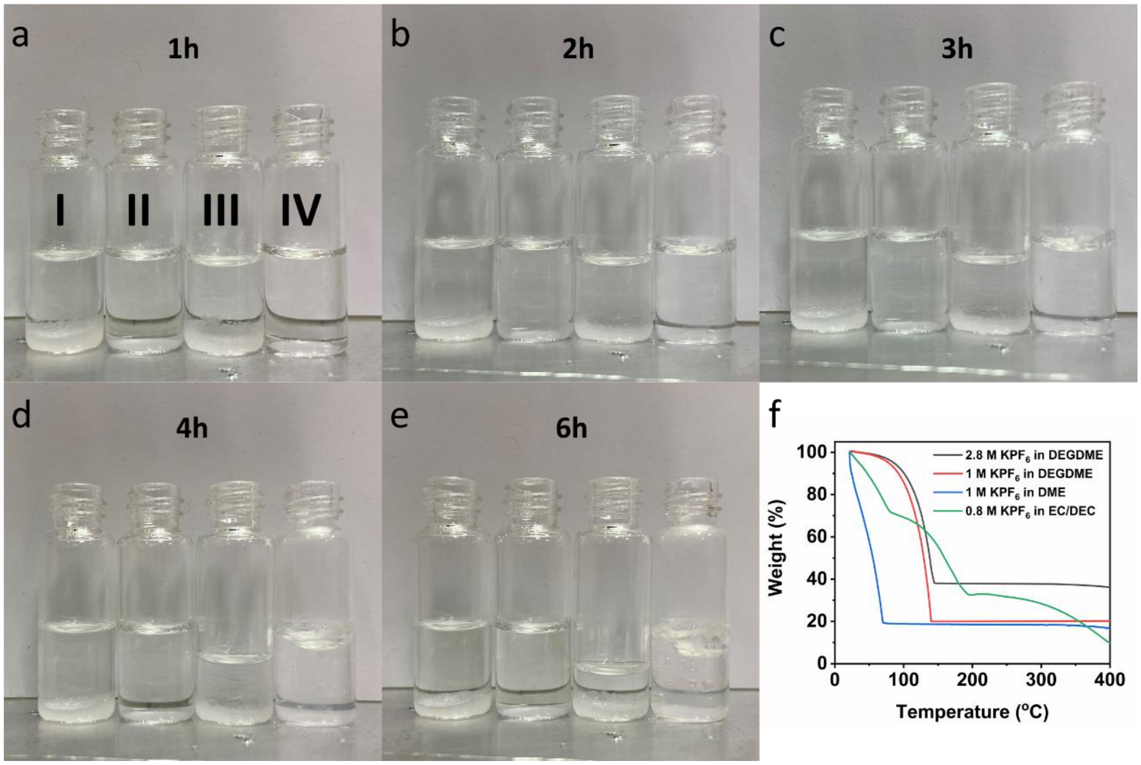

Figure S25. (a-e) Different electrolytes heated to 100°C at different time periods. (I) 2.8M KPF_6 in DEGDME, (II) 1M KPF_6 in DEGDME, (III) 1M KPF_6 in DME and (IV) 0.8M KPF_6 in EC/DEC. (f) TGA thermograms of different electrolytes.

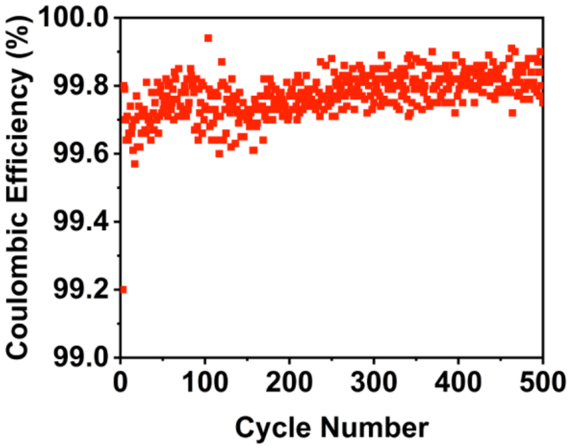

Figure S26. Coulombic efficiency of the all-organic RPB at 1 A g^{-1} and 80°C.

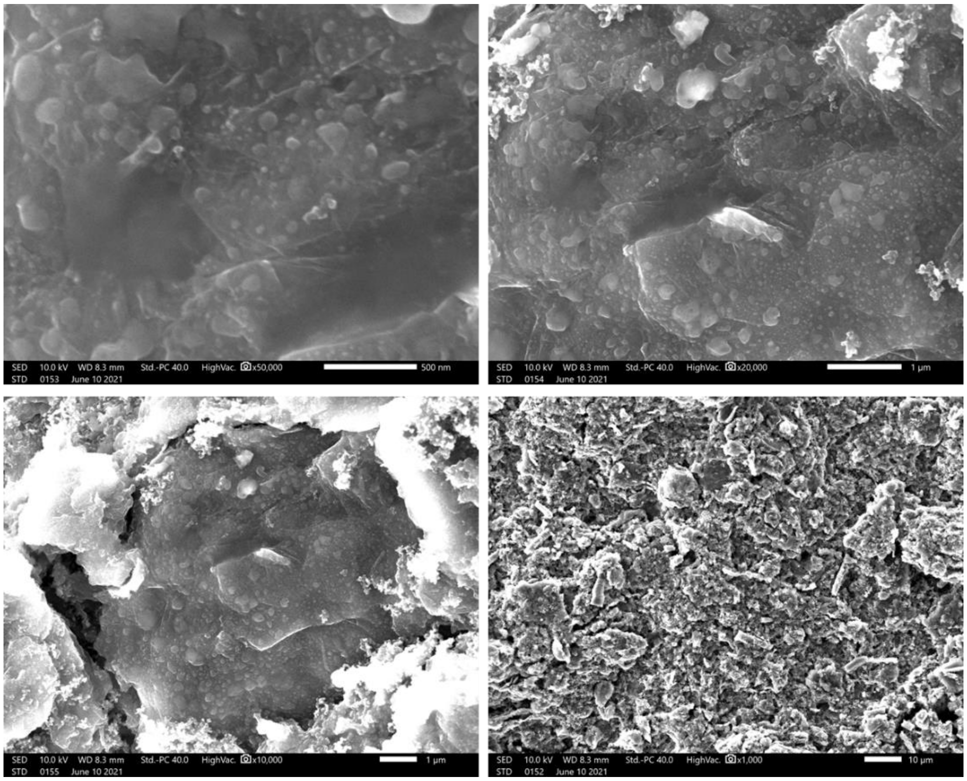

Figure S27. SEM images of the TBPS/NG anode after 20 cycles at 200 mA g^{-1} and 70°C.

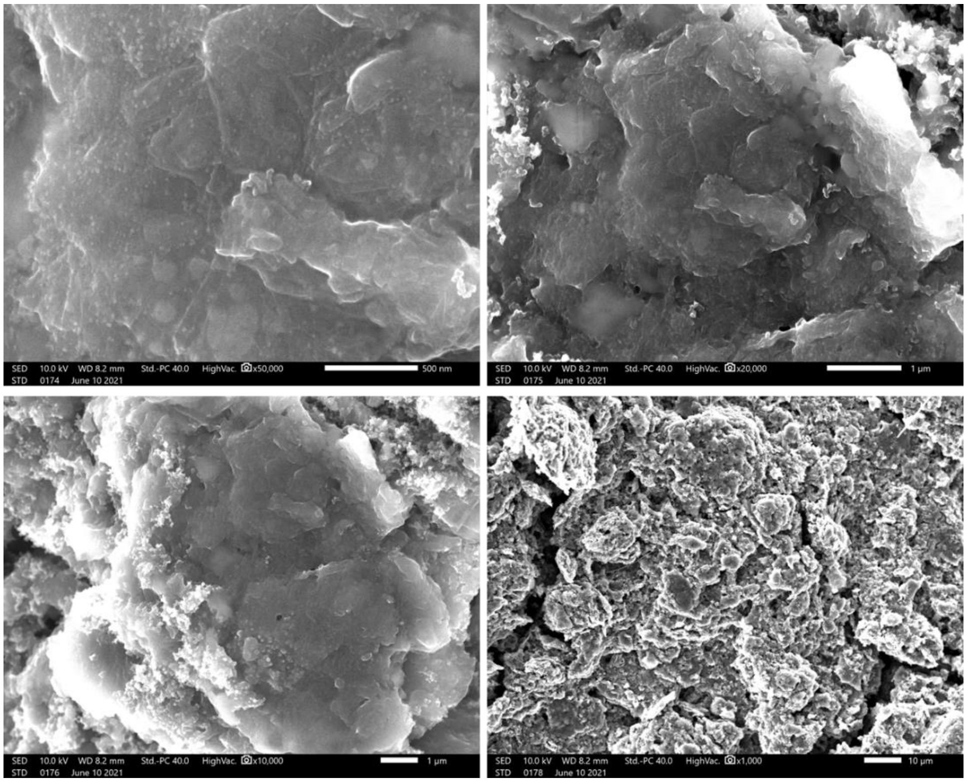

Figure S28. SEM images of the TBPS/NG anode after 20 cycles at 200 mA g^{-1} and 80°C.

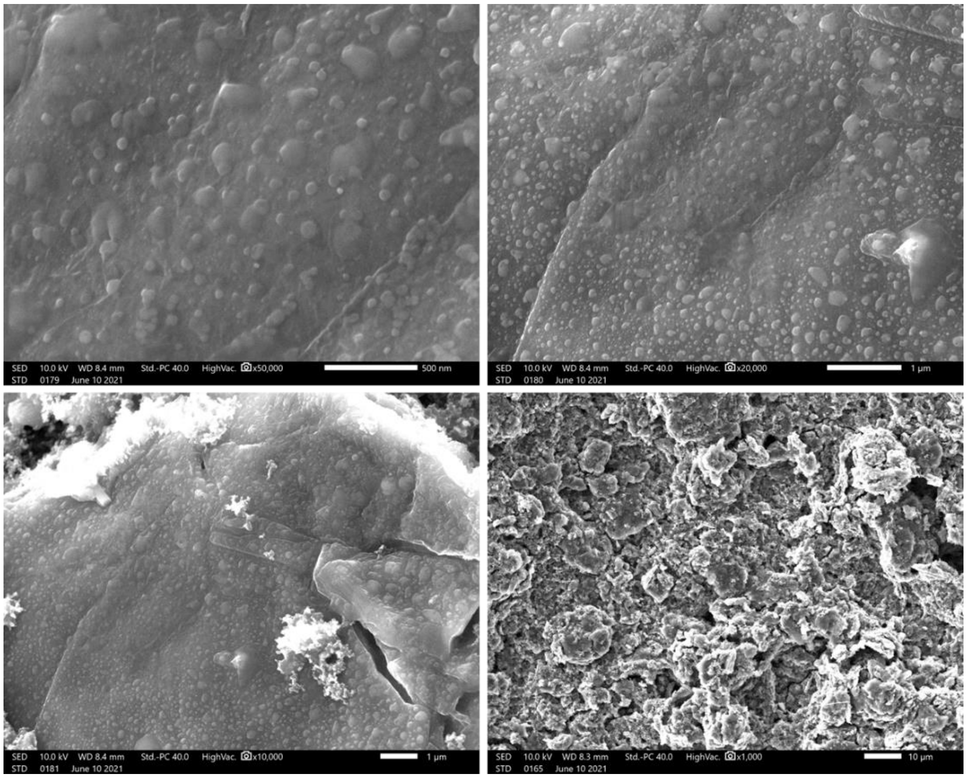

Figure S29. SEM images of the TBPS/NG anode after 20 cycles at 200 mA g^{-1} and 90°C.

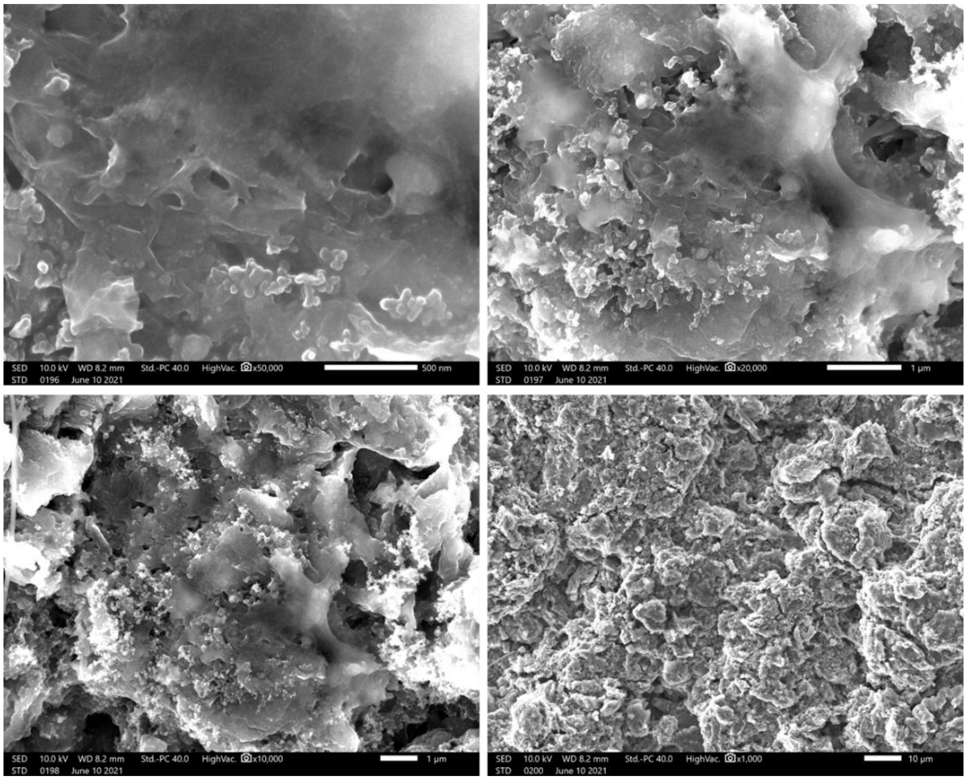

Figure S30. SEM images of the TBPS/NG anode after 20 cycles at 200 mA g^{-1} and 100°C.

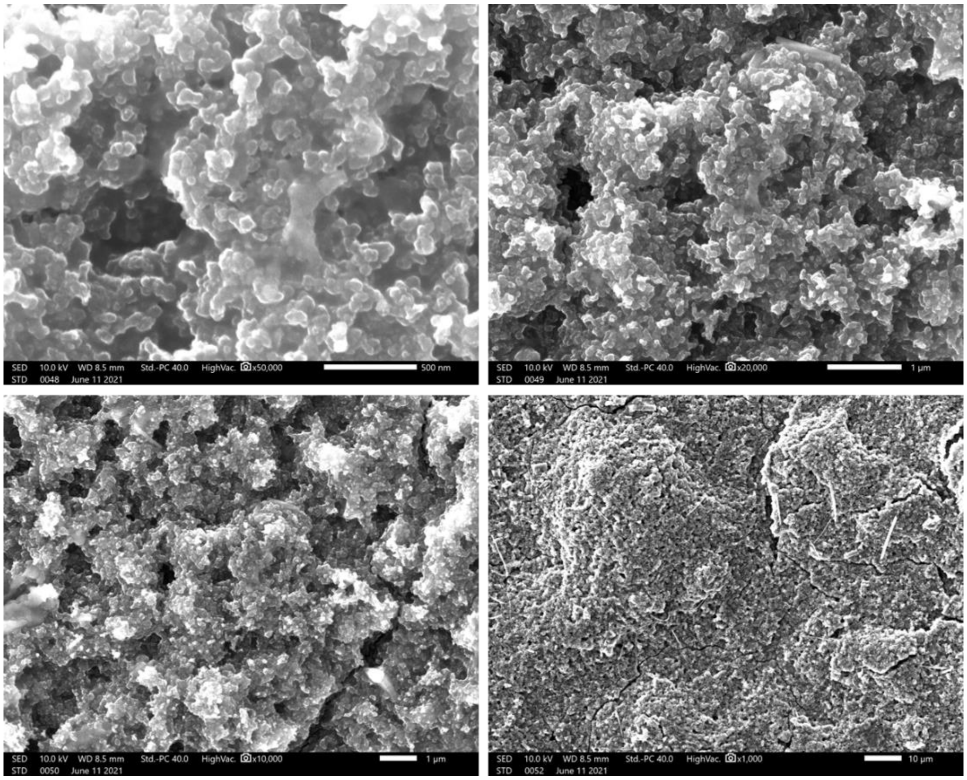

Figure S31. SEM images of the TBPS anode after 20 cycles at 200 mA g^{-1} and 70°C.

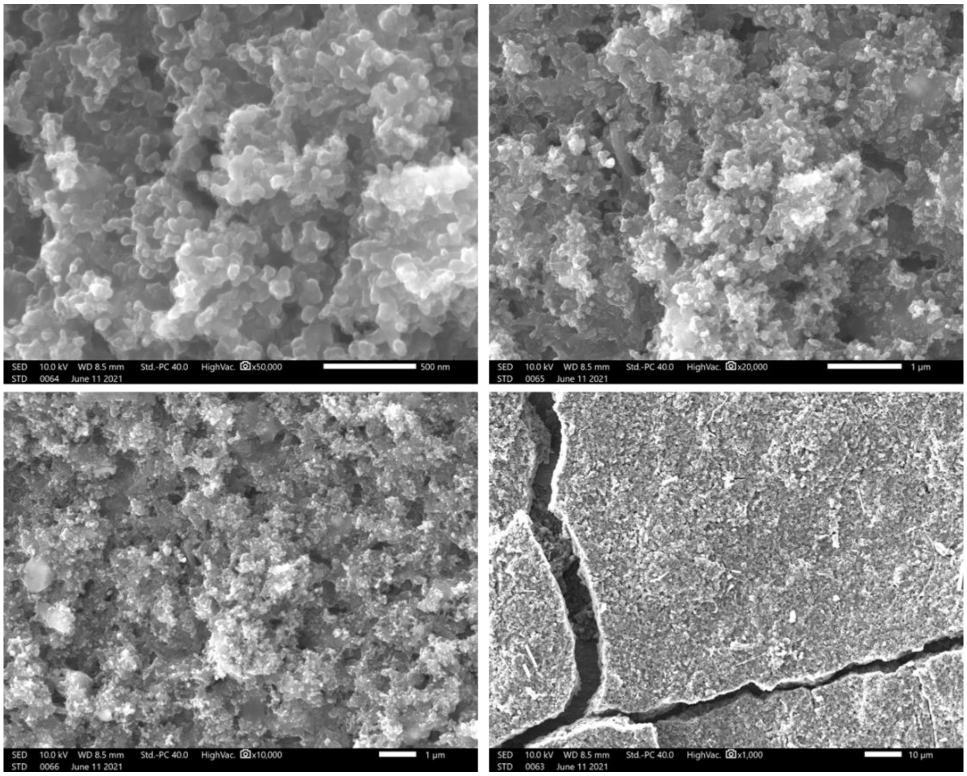

Figure S32. SEM images of the TBPS anode after 20 cycles at 200 mA g^{-1} and 80°C.

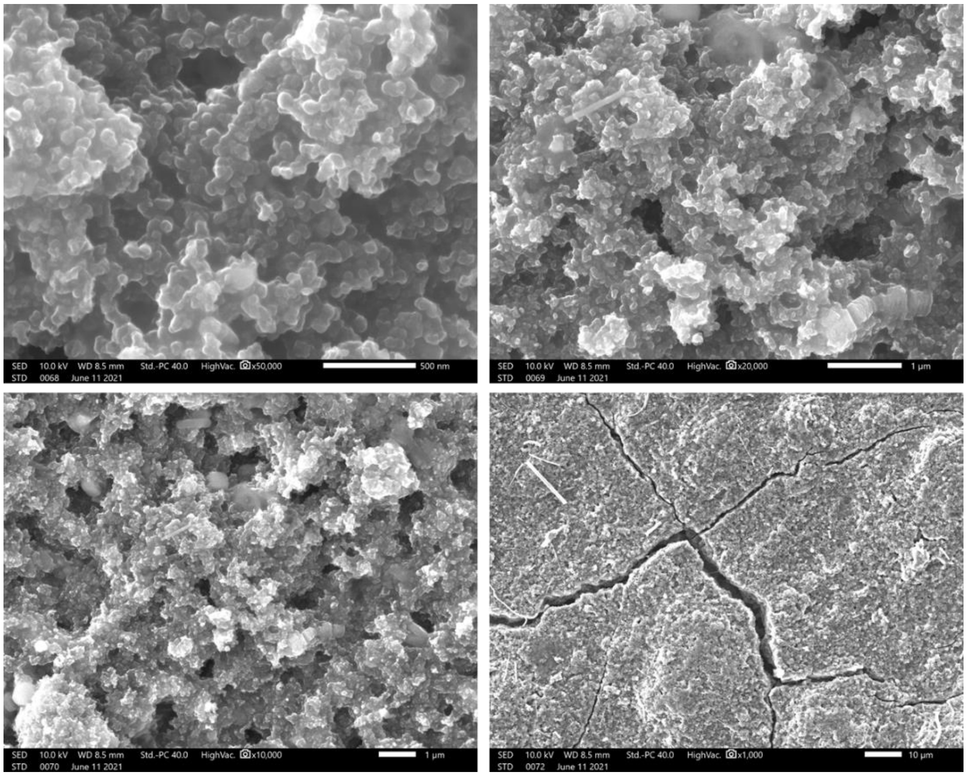

Figure S33. SEM images of the TBPS anode after 20 cycles at 200 mA g^{-1} and 90°C.

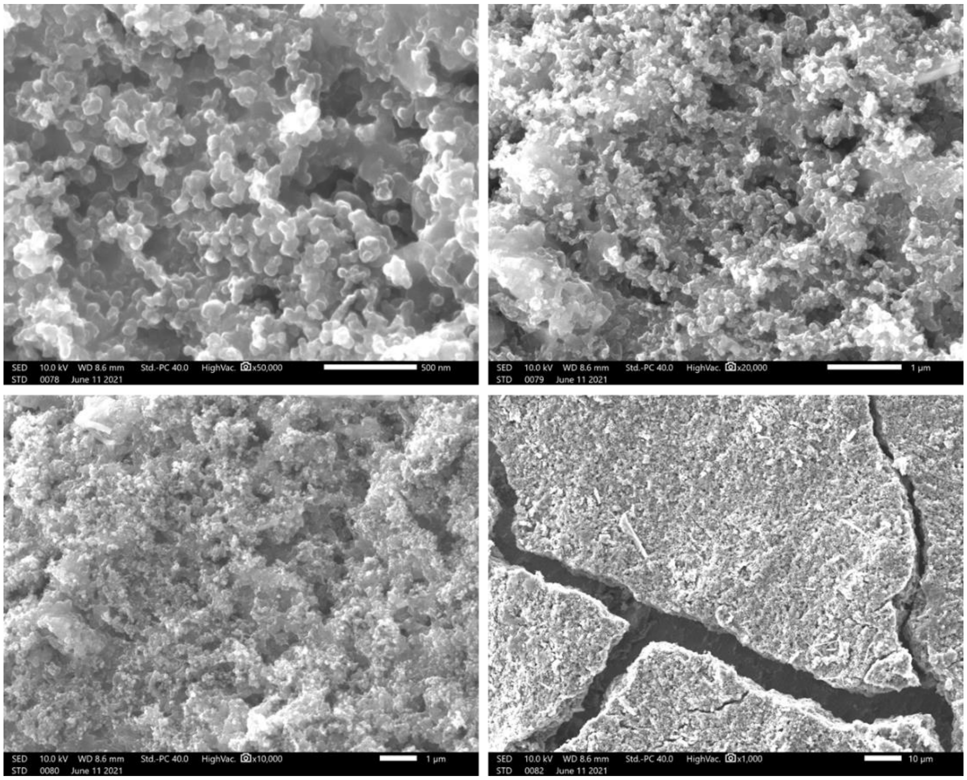

Figure S34. SEM images of the TBPS anode after 20 cycles at 200 mA g^{-1} and $100^{o}C$.

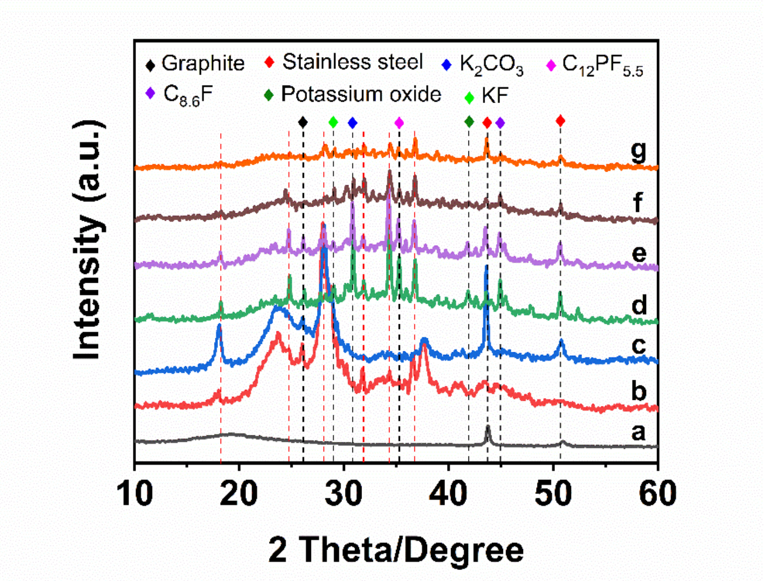

Figure S35. XRD patterns of the thick TBPS/NG anode in the all-organic RPB before and after cycling at different temperatures. (a) stainless steel mesh, (b) the TBPS/NG powder, (c) the pristine TBPS/NG anode; The TBPS/NG anode after 20 cycles under current density of 200 mA g^{-1} at (d) 70°C, (e) 80°C, (f) 90°C, and (g) 100°C.

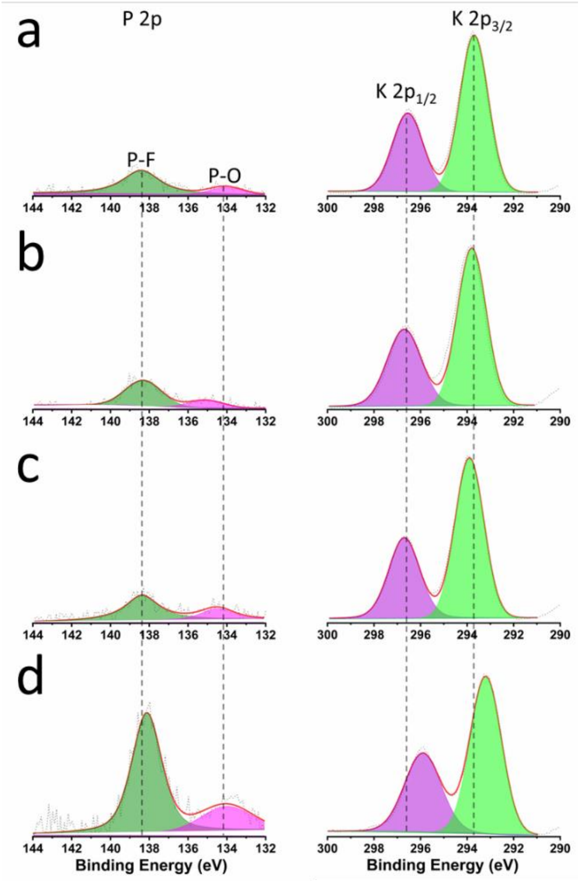

Figure S36. P 2p and K 2p XPS spectra of the TBPS/NG anode after 20 cycles under the current density of 200 mA g^{-1} at (a) 70°C, (b) 80°C, (c) 90°C, and (d) 100°C.

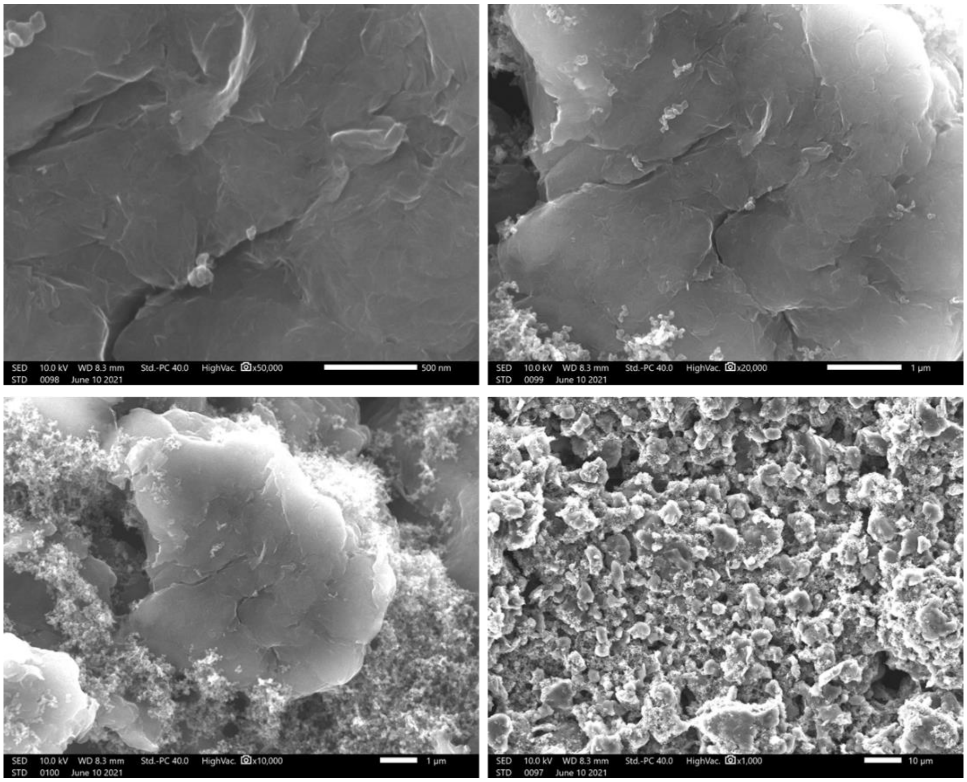

Figure S37. SEM images of the pristine PANI/NG cathode.

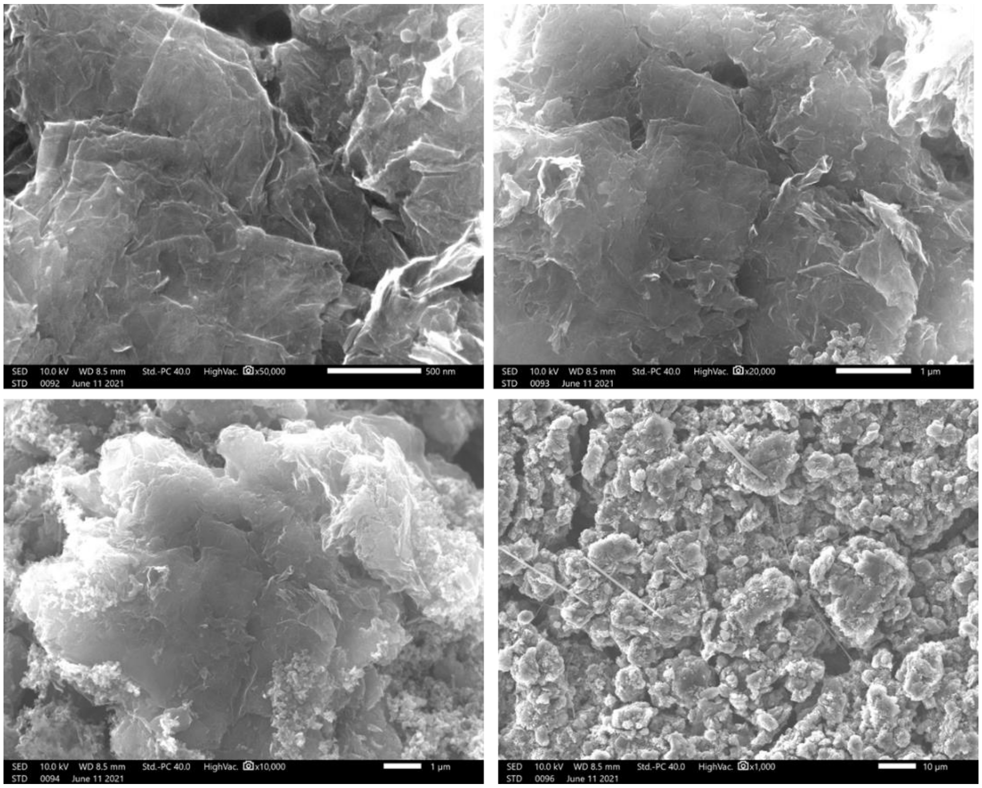

Figure S38. SEM images of the PANI/NG cathode after 20 cycles at 200 mA g^{-1} and $70^{o}C$.

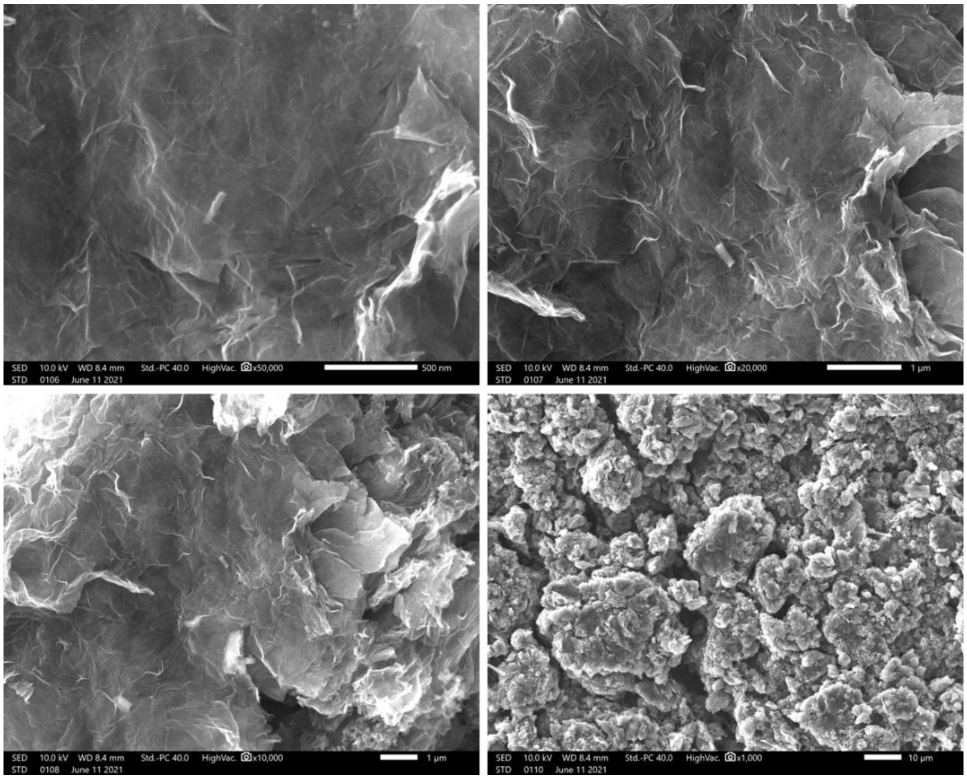

Figure S39. SEM images of the PANI/NG cathode after 20 cycles at 200 mA g^{-1} and $80^{o}C$.

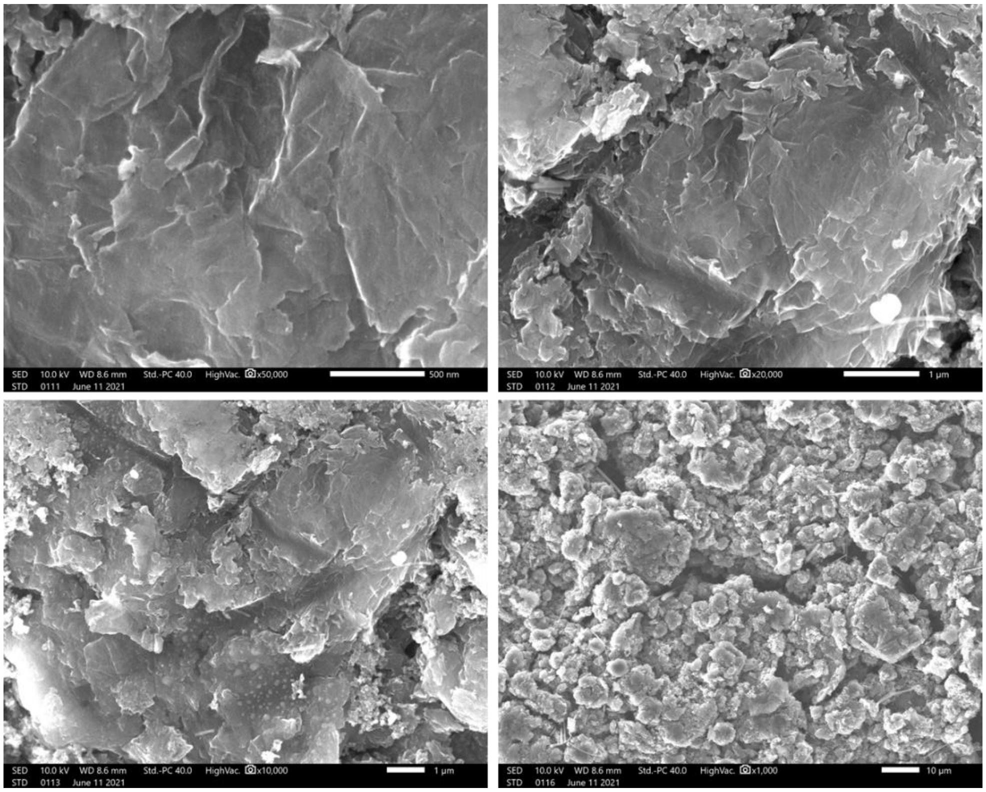

Figure S40. SEM images of the PANI/NG cathode after 20 cycles at 200 mA g^{-1} and 90°C.

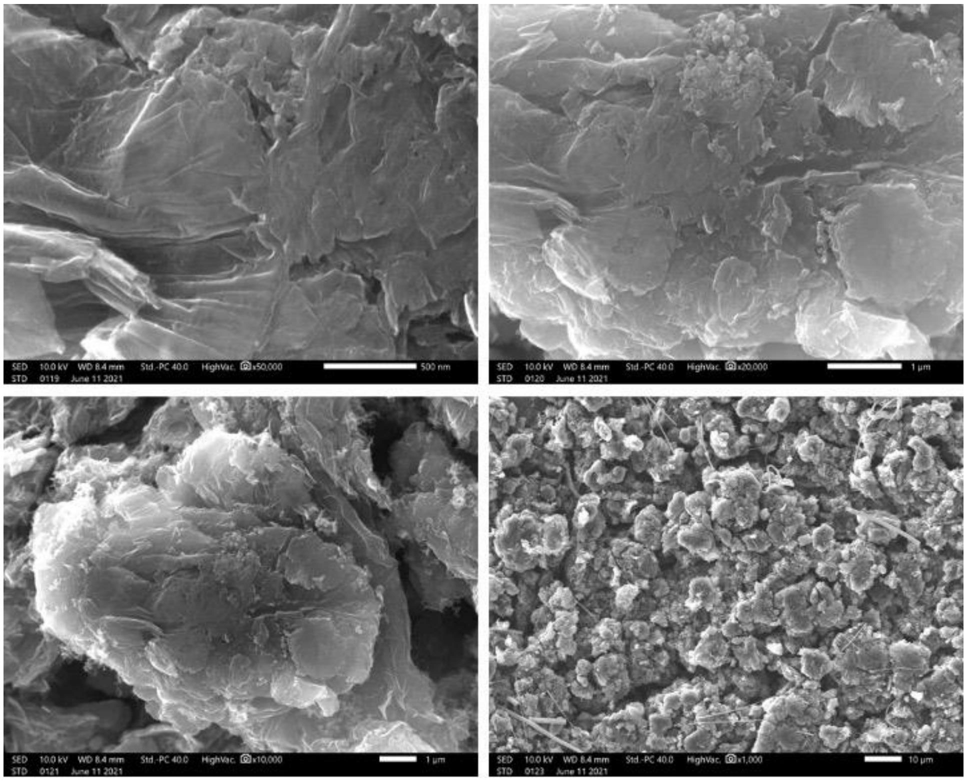

Figure S41. SEM images of the PANI/NG cathode after 20 cycles at 200 mA g^{-1} and 100°C.

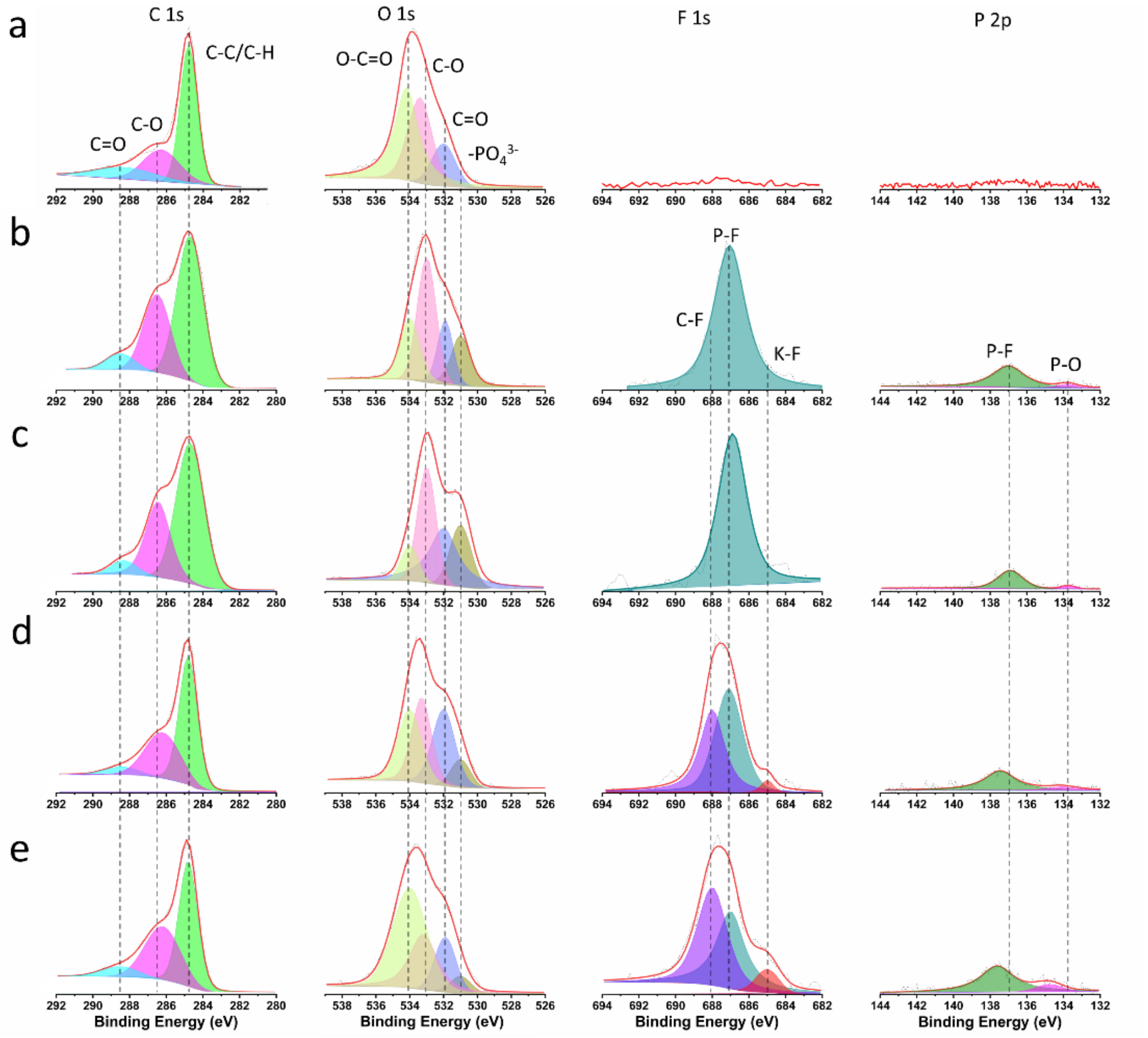

Figure S42. XPS spectra of (a) the pristine PANI/NG cathode and the PANI/NG cathodes after 20 cycles under the current density of 200 mA g^{-1} at (b) 70°C, (c) 80°C, (d) 90°C, and (e) 100°C.
